# Annotated type catalogue of the Orthalicoidea (Mollusca, Gastropoda) in the Royal Belgian Institute of Sciences, Brussels, with descriptions of two new species

**DOI:** 10.3897/zookeys.101.1133

**Published:** 2011-05-31

**Authors:** Abraham S.H. Breure

**Affiliations:** Netherlands Centre for Biodiversity Naturalis, P.O. Box 9517, Leiden, the Netherlands

**Keywords:** Bolivia, Amphibulimidae, Bulimulidae, Bothriembryontidae, Megaspiridae, Orthalicidae, Simpulopsidae, types, biohistory

## Abstract

The type status is described of 57 taxa from the superfamily Orthalicoidea in the collection of the Brussels museum. Two new species are described: *Stenostylus perturbatus* **sp. n.**, and *Suniellus adriani* **sp. n.** New lectotypes are designated for *Bulimulus (Naesiotus) amastroides* Ancey, 1887; *Bulimulus blanfordianus* Ancey, 1903; *Bulimulus montivagus chacoensis* Ancey, 1897; *Bulimus coloratus* Nyst, 1845; *Plecochilus dalmasi* Dautzenberg, 1900; *Placostylus porphyrostomus elata* Dautzenberg, 1923; *Bulimulus ephippium* Ancey, 1904; *Bulimus fulminans* Nyst, 1843; *Bulimus funckii* Nyst, 1843; *Orphnus thompsoni lutea* Cousin, 1887; *Bulimus melanocheilus* Nyst, 1845; *Orphnus thompsoni nigricans* Cousin, 1887; *Orphnus thompsoni olivacea* Cousin, 1887; *Bulimulus pollonerae* Ancey, 1897; *Orphnus thompsoni zebra* Cousin, 1887. New combinations are: *Bostryx borellii* (Ancey, 1897); *Bostryx carandaitiensis* (Preston, 1907); *Protoglyptus mazei* (Crosse, 1874); *Kuschelenia (Vermiculatus) sanborni* (Haas, 1947). New synonymies are established for the following nominal taxa: *Orphnus thompsoni* var. *lutea* Cousin, 1887 = *Kara thompsonii* (Pfeiffer, 1845); *Orphnus thompsoni* var. *nigricans* Cousin, 1887 = *Kara thompsonii* (Pfeiffer, 1845); *Thaumastus nystianus* var. *nigricans* Cousin, 1887 = *Drymaeus (Drymaeus) nystianus* (Pfeiffer, 1853); *Orphnus thompsoni* var. *olivacea* Cousin, 1887 = *Kara thompsonii* (Pfeiffer, 1845); *Orphnus thompsoni* var. *zebra* Cousin, 1887 = *Kara thompsonii* (Pfeiffer, 1845).

## Introduction

The Orthalicoidea is a dominant faunal element in the Neotropics (Breure and Mogollón 2010), but also has a number of genera with a Gondwanan distribution (Breure 1979; Herbert and Mitchell 2009; Neubert et al. 2009). The relationships within this group have predominantly been based on morphological data (Breure 1974b, 1979, Breure and Schouten 1985), but are being re-defined by ongoing molecular work (Breure et al. 2010; Breure and Romero in preparation). In this superfamily approximately 1750 taxon names are available, which calls for an inventory of as much type material as possible to enable revisionary work. This paper complements previous data on type material for this group in the museums of Paris (Breure 1975b), Zürich (Breure 1976), Frankfurt (Zilch 1971; Neubert and Janssen 2004), Berlin (Köhler 2007), and—forthcoming—in the London museum (Breure and Ablett, unpublished data). The aim of this paper is to present data on the types of Orthalicoidea in the collection of the Royal Belgian Institute of Sciences (hereafter RBINS), Brussels.

## The collection

One of the interesting parts of the RBINS malacological collection is the former collection of Philippe Dautzenberg (1849–1935; see Lamy 1935 and Duchamps 1999 for a biography). This collection is a rich source for many groups, as Dautzenberg was very keen on the quality of his acquisitions (either by exchange or purchase), and always aimed to have species represented by larger series to allow study of the variation. He befriended many malacologists of his time and thanks to ample financial resources was able to acquire important shell collections whenever they came on the market. In this way, parts of the Ancey collection (see Wood and Gallichan 2008), and the Cousin collection (see below), are now housed in RBINS.

**Figure 1.**
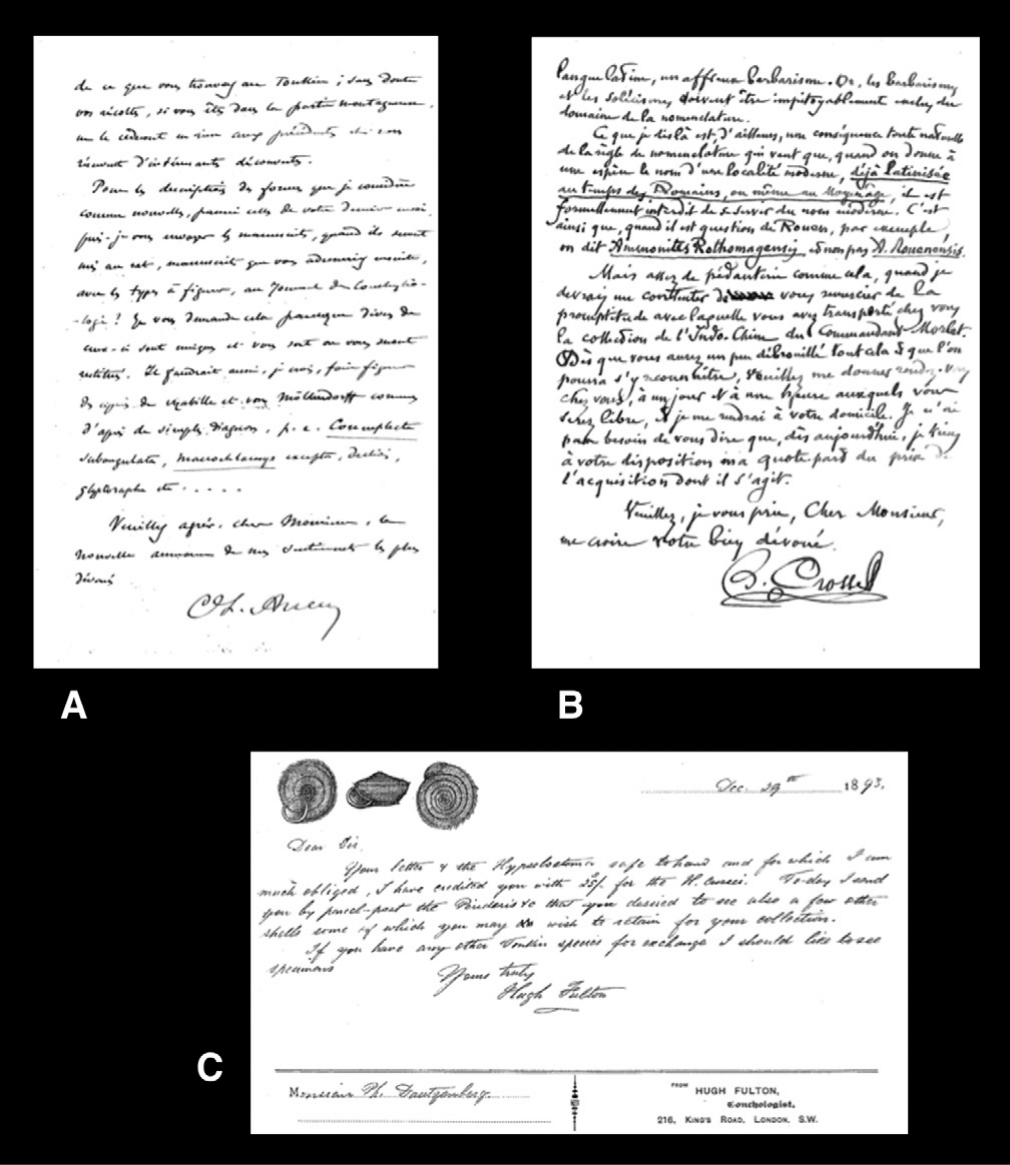
Excerpts of autographs from the Dautzenberg archive. **A** C.F. Ancey. **B** H. Crosse. **C** H.C. Fulton.

**Figure 2.**
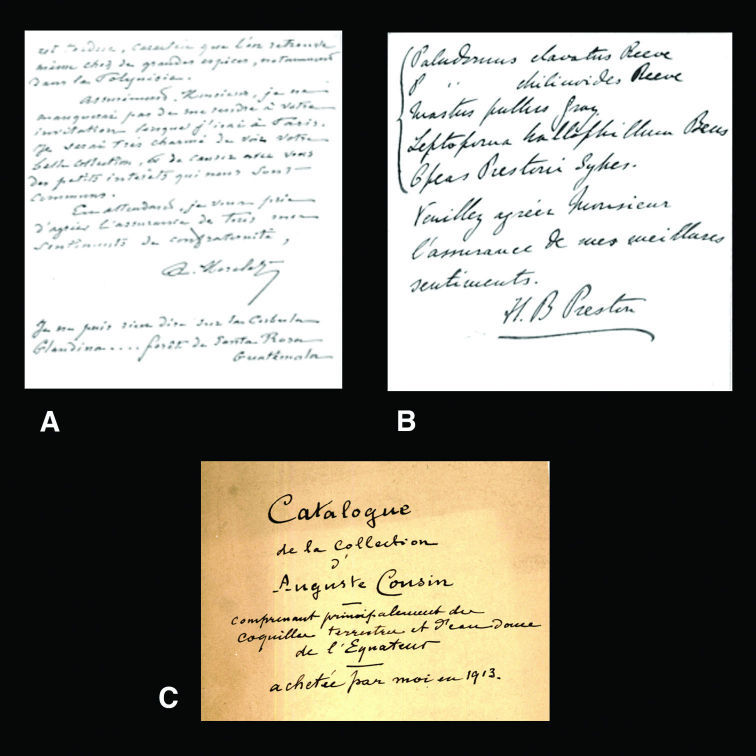
**A–B** Excerpts of autographs from the Dautzenberg archive. **A** A. Morelet. **B** H.B. Preston. **C** Cover of the inventory of the collection of A. Cousin, in Dautzenberg’s handwriting.

Duchamps (1999: 3–19) published an extensive list of collaborators and sources of material for Dautzenberg. In the Dautzenberg archive, autographs were found of the following persons mentioned in this paper, which are relevant to compare handwriting on labels in the Dautzenberg collection: César-Marie-Félix Ancey, parts of whose collection Dautzenberg purchased (Wood and Gallichan 2008); Hugh Coomber Fulton, shell dealer who sold type material to Dautzenberg; Arthur Morelet, who donated several types; Hugh Berthon Preston, also a shell dealer, and well-known for the distribution of much material under manuscript names (see also Adam 1971); Hermann Rolle, another shell dealer of whom Dautzenberg was a client. Excerpts of these autographs are given in Figs 1, 2A–B; examples of labels in their label handwriting may be found in different figures, of taxa related to them. It may be noted, that after the receipt of the Dautzenberg collection by RBINS, some type material has been added through exchange of specimens originally in the Dautzenberg collection from other sources (Van Goethem, pers. commun.).

Two other authors need mentioning, who were not referred to by Duchamps (1999). The first is Auguste Cousin, a Frenchman who lived for many years in Ecuador and travelled extensively throughout the country. Although he may be regarded as the “father of Ecuadorian malacology” nothing is known about his life, except that he was born in Ecuador in 1835 and died in Paris, France in 1899 (Correoso, pers. commun.). He published only one, extensive paper on the non-marine malacofauna of that country (Cousin 1887). Through his relationship with Jousseaume, some material was known to exist in the MNHN collection (Breure 1975b). To my surprise, there was ample material in the Dautzenberg collection. An inventory of the Cousin collection was found by Rose Sablon, RBINS technician, in the Dautzenberg archive. It appeared that Dautzenberg acquired this collection in 1913 (Fig. 2C), but it is not documented from whom.

The second author is Pierre-Henri Nyst, a Belgian paleontologist who in his youth described several species of non-marine molluscs from South America, supplied by Belgian explorers (Dupont 1882: 314). His type material, for many years thought to be lost, was now found in the RBINS collection.

## Methods

The following criteria were applied to assess potential type material: (a) the locality fits with the original description (taking into account changes in geographical names due to political or administrative reasons); (b) alleged type material is in accordance with the established understanding of the taxon. In order to fulfill the requirements of article 74 of the International Code of Zoological Nomenclature (ICZN), any lectotype designations herein are to be understood as to have the sole purpose of fixing the status of these specimens as the sole name-bearing types of the nominal taxa, to ensure the name’s proper and consistent application, even if this is not explicitly done in every single case but abbreviated as “lectotype designation”. Lectotypes are designated herein using the following criteria, in order of preference: (1) the relevant specimen was figured in the original description, or in subsequent revisionary works; (2) if no original figure was published, a specimen was selected that matches as closely as possible the measurements given in the original description.

For each taxon, the original publication—in which the taxon was proposed—is mentioned, as well as papers in which reference is made to the type material. The type locality is quoted from the original publication in the original wording and language, with clarifying notes between square brackets. The name of the collector, if given in the original paper, is only mentioned (in italics) if it might give a clue about the type status of material present in the collection. The text of the original, or oldest, label is quoted, together with information from subsequent labels if containing information necessary for a correct interpretation. All labels have been photographed and are figured for future historic reference. The dimensions of the type are quoted, as given in the original paper. Dimensions of the type specimens have been taken with a digital caliper, using the methods figured by Breure (1974a: figs 2–3); measurements up to 10 mm have an accuracy of 0.1 mm, those above 10 mm are accurate to 0.5 mm. Due to improvements in accuracy of Vernier calipers, the measurements given herein are in several cases slightly different from those originally reported. In the case of syntypes, only the largest specimen has been measured. Under type material the RBINS-registration numbers are given; if specimens from different localities are present, the order of the lots corresponds with the information of the different labels. The number of specimens originally available, if quoted by the original author, is mentioned under remarks. Further remarks are given to describe any individual characteristics of the type specimens or any other details of the type lot. The current systematic position is given, following the generic scheme of Breure (1979) and the familial arrangement of Breure et al. (2010) and Breure and Romero (in preparation).

Abbreviations used for depositories of material are: ANSP, Academy of Natural Sciences, Philadelphia, U.S.A.; FMNH, Field Museum of Natural History, Chicago, U.S.A.; MHNG, Muséum d’Histoire Naturelle, Genève, Switzerland; MNHN, Museum national d’Histoire naturelle, Paris, France; NHM, Natural History Museum, London, U.K.; RMNH, Nederlands Centrum voor Biodiversiteit Naturalis (formerly Rijksmuseum van Natuurlijke Historie), Leiden, Netherlands; SMF, Natur-Museum Senckenberg, Frankfurt am Main, Germany; UF, Florida State Museum, Gainesville, U.S.A.; ZMB, Zoologisches Museum, Humboldt Universität, Berlin, Germany. Other abbreviations used are: /, end of line in cited text; coll., collection; D, shell diameter; H, shell height; leg., *legit*, collected; MT, type collection of RBINS Malacology Section; W, number of whorls.

## Systematics

### Systematic list of nominal taxa arranged in generic order

This systematic list follows the arrangement of families as proposed by Breure et al. (2010) and Breure and Romero (in preparation), and the generic order from Breure (1979). Within the families, genera are presented in alphabetical order. Neubert and Janssen (2004) correctly stated that the system of Orthalicoidea still remains in an unsatisfactory state. This is partly due to the *sensu lato* approach followed for some genera (notably *Bostryx* and *Naesiotus*) by Breure (1979), which caused a considerable number of taxa to be affiliated with a relatively low number of genera. The phylogenetic studies of Breure et al. (2010) and Breure and Romero (in preparation) resolve this only partly. As some revisionary work, including phylogenetic studies, related to the status of these genera is ongoing, their *sensu lato* status is here tentatively retained with the exception of the status of *Protoglyptus*. This taxon, considered a synonym of *Naesiotus* by Breure (1979), is herein treated generic status given its separate position in the analysis of Breure and Romero (in preparation). Also the status of *Kara*, treated as a subgenus of *Thaumastus* by Breure (1979), is now changed on the basis of their analysis; this taxon is now given generic status. No phylogenetic data have been obtained yet for some other genera treated in this paper (e.g. *Dryptus*); their familial relationship remains tentative until a more satisfactory arrangement can be presented. Finally, re-interpreting the results of (Breure (1978, 1979), the genus *Scutalus* is now restricted to *Scutalus (Scutalus)* sensu Breure (1979). Two other subgenera, *Kuschelenia* and *Vermiculatus* are now considered as *Kuschelenia (Kuschelenia)* and *Kuschelenia (Vermiculatus)* respectively; *Suniellus* is herein given generic rank.

At the family level the new scheme of Breure and Romero (in preparation) is followed. They give familial rank to the tribus Simpulopsini Schileyko, 1999, and showed that the Placostylidae Pilsbry, 1946 *sensu* Neubert et al. (2009) fall within the Bothriembryontidae Iredale, 1937.

**Family Amphibulimidae P. Fischer, 1873**

*Dryptus* Albers, 1860

*Dryptus funckii* Nyst, 1843

*Plekocheilus* (*Eurytus*) Albers, 1850

*Plekocheilus (Eurytus) coloratus* Nyst, 1845; *Plekocheilus (Eurytus) dalmasi* Dautzenberg, 1900

*Plekocheilus* (*Plekocheilus*) Guilding, 1828

*Plekocheilus (Plekocheilus) fulminans* Nyst, 1845

**Family Orthalicidae Martens, 1860**

*Kara* Strebel, 1910

*Kara lutea* Cousin, 1887; *Kara nigricans* Cousin, 1887; *Kara olivacea* Cousin, 1887; *Kara zebra* Cousin, 1887

**Family Megaspiridae Pilsbry, 1904**

*Thaumastus* Albers, 1860

*Thaumastus blanfordianus* Ancey, 1903

**Family Bothriembryontidae Iredale, 1937**

*Aspastus* Albers, 1850

*Aspastus porphyrochila* Dautzenberg and Bernier, 1901

*Placostylus* Beck, 1837

*Placostylus auriculatus* Dautzenberg and Bouge, 1923; *Placostylus elatus* Dautzenberg, 1923; *Placostylus paraspiritus* A.W.B. Powell, 1951; *Placostylus whareana* A.W.B. Powell, 1951

**Family Simpulopsidae Schileyko, 1999**

*Rhinus* Albers, 1860

*Rhinus argentinus* Ancey, 1901

*Simpulopsis* (*Eudioptus*) Albers, 1860

*Simpulopsis (Eudioptus) ephippium* Ancey, 1904

**Family Bulimulidae Tryon, 1867**

*Bostryx* Troschel, 1847

*Bostryx alausiensis* Cousin, 1887; *Bostryx albicolor* Morelet, 1863; *Bostryx baeri* Dautzenberg, 1901; *Bostryx bonneti* Ancey, 1902; *Bostryx borellii* Ancey, 1887; *Bostryx carandaitiensis* Preston, 1907; *Bostryx emaciatus* Morelet, 1863; *Bostryx huayaboensis* Dautzenberg, 1901; *Bostryx iocosensis* Dautzenberg, 1901; *Bostryx juana* Cousin, 1887; *Bostryx metagyra* Pilsbry and Olsson, 1949; *Bostryx moniezi* Dautzenberg, 1901; *Bostryx perforatus* Haas, 1951; *Bostryx spiculatus* Morelet, 1860; *Bostryx veruculum* Morelet, 1860

*Drymaeus* (*Drymaeus*) Albers, 1850

*Drymaeus (Drymaeus) abruptus* Rolle, 1904; *Drymaeus (Drymaeus) colimensis* Rolle, 1895; *Drymaeus (Drymaeus) icterica* Ancey, 1892; *Drymaeus (Drymaeus) jousseaumei* Dautzenberg, 1901; *Drymaeus (Drymaeus) nigricans* Cousin, 1887; *Drymaeus (Drymaeus) scolioides* Dautzenberg, 1901; *Drymaeus (Drymaeus) solidus* Preston, 1907

*Drymaeus* (*Mesembrinus*) Albers, 1850

*Drymaeus (Mesembrinus) interruptus* Preston, 1909; *Drymaeus (Mesembrinus) pallidus* Preston, 1909

*Naesiotus* Albers, 1850

*Naesiotus albemarlensis* Dall, 1917; *Naesiotus amastroides* Ancey, 1887; *Naesiotus bizonalis* Ancey, 1887; *Naesiotus chacoensis* Ancey, 1897; *Naesiotus cucullinus* Dall, 1917; *Naesiotus duncanus* Dall, 1893; *Naesiotus gilderoyi* Van Mol, 1972; *Naesiotus lycodus* Dall, 1917; *Naesiotus pollonerae* Ancey, 1897

*Protoglyptus* Pilsbry, 1897

*Protoglyptus dejectus* Fulton, 1901; *Protoglyptus mazei* Crosse, 1874

*Rabdotus* Albers, 1850

*Rabdotus hesperius* Pilsbry and Ferriss, 1924

*Kuschelenia* (*Vermiculatus*) Breure, 1978

*Kuschelenia (Vermiculatus) sanborni* Haas, 1947

## Alphabetic list of taxa by species name

### Bulimulus (Drymaeus) abruptus

Rolle, 1904

http://species-id.net/wiki/Bulimulus_(Drymaeus)_abruptus

Figs 12A, 12i

Bulimulus (Drymaeus) abruptus Rolle 1904: 35.

#### Type locality.

“Huancabamba in Peru”; see remarks.

#### Label.

“Huancabamba, / Peru 1904”; in Rolle’s handwriting. Another label, in Dautzenberg’s handwriting, indicating that he obtained it on 16.vii.1907 from Rolle.

#### Dimensions.

“Alt. 44, diam. max. 24 (..) mm”; figured specimen H 36.6, D 16.9, W 6.5.

#### Type material.

RBINS/MT2332, two syntypes (Dautzenberg coll.).

#### Remarks.

Rolle did not state on how many specimens his description was based upon. However, there is another specimen in the NHM collection which will be designated lectotype (Breure and Ablett, unpublished data). The locality is ambiguous, as there are several places called “Huancabamba” in Peru. There were, however, several other species described by Rolle from the same locality, among them *Columbinia huancabambensis*, which is regarded by Loosjes and Loosjes-van Bemmel (1984: 33) as occurring in northern Peru. Another species described in the same paper, *Systrophia moellendorffii*, was said by Haas (1955: 367) to have been rediscovered in the Chanchamayo valley [Dept. Pasco]. These, and the fact that a third Rolle species, *Newboldius illustris* is known to occur in the same region, makes Dept. Pasco, Huancabamba likely to be the locality where Rolle’s taxa occur.

#### Current systematic position.

Bulimulidae, *Drymaeus (Drymaeus) abruptus* (Rolle, 1904).

### Thaumastus alausiensis

Cousin, 1887

http://species-id.net/wiki/Thaumastus_alausiensis

Figs 10A–B, 10i

Thaumastus alausiensis Cousin 1887: 228, pl. 4 fig. 13.
Peronaeus (Lissoacme) alausiensis  (Cousin); Breure 1975b: 1141, pl. 6 fig. 4 (lectotype 	designation).
Bostryx alausiensis  (Cousin); Breure 2008: 244, figs 1–5.

#### Type locality.

[Ecuador] “sur le versant du mont Hacu, entre Achapallas et la rivière Sula, sur le territoire Alausi, province de Chimborazo”.

#### Label.

“Racu, descente de Achupalla / au Rio Sula”, in Cousin’s handwriting.

#### Dimensions.

“long. 25 mm; diam. 9 à 10 mm”; figured specimen H 25.2, D 11.0, W 8.2.

#### Type material.

RBINS/MT2333, paralectotype; MT2334, 12 paralectotypes (partly juvenile), Cousin leg. (Dautzenberg coll.).

#### Remarks.

The material which Breure (1975) used to select a lectotype, came from the Jousseaume collection and originated from Cousin. However, the locality was imprecise (“Équateur” [Ecuador]). At that time, no other material from Cousin was known. In RBINS, material originating from Cousin and exactly corresponding to the type locality has been found. Therefore it may now be questioned if the Jousseaume material was validly regarded as the prime type material. However, in the inventory of Cousin’s collection, a total of 30 specimens is mentioned and it cannot be excluded that Jousseaume’s material originated from the Cousin collection.

#### Current systematic position.

Bulimulidae, *Bostryx alausiensis* (Cousin, 1887).

### Bulimulus (Naesiotus) albemarlensis

Dall, 1917

http://species-id.net/wiki/Bulimulus_(Naesiotus)_albemarlensis

Bulimulus (Naesiotus) albemarlensis Dall 1917: 377; Dall and Ochsner 1928: 167, pl. 8 figs 7–8; Boss et al. 1968: 17.
Naesiotus albemarlensis  (Dall); Breure 1979: 67; Köhler 2007: 136, fig. 45.

#### Type locality.

[Ecuador, Galápagos, Isla Isabela] “near [Puerto] Villamil at 2300 to 3300 feet elevation”.

#### Label.

“Near Villamil / Albemarle Isl., Galapagos” on label of Stanford University.

#### Dimensions.

“Length of shell 15, (...) diameter 9 mm”; largest specimen H 13.6, D 9.0, W 5.6.

#### Type material.

RBINS/MT1911, two paratypes, Ochsner leg.

#### Remarks.

The material was donated by H.G. Schenk. For data on the role of Schenck and the relationship between Stanford University and RBINS, see Keen (1980).

#### Current systematic position.

Bulimulidae, *Naesiotus albemarlensis* (Dall, 1917).

### Bulimus albicolor

Morelet, 1863

http://species-id.net/wiki/Bulimus_albicolor

Figs 11I, 11ix

Bulimus albicolor Morelet 1863: 199, pl. 11 fig. 9.
Bostryx albicolor  (Morelet); Breure 1979: 51.

#### Type locality.

[Peru, Dept. Ayacucho] “Huanta et de la vallée de l’Apurimac”.

#### Label.

“Pérou”; taxon name in Morelet’s handwriting, locality data in Dautzenberg’s handwriting (“ex auctore”).

#### Dimensions.

“Longit 28, diam. 9 mm”; figured specimen H 22.2, D 9.76, W 5+.

#### Type material.

RBINS/MT2335, one syntype, ex Morelet (Dautzenberg coll.).

#### Remarks.

Additional syntype material is present in the MHNG and NHM collections.

#### Current systematic position.

Bulimulidae, *Bostryx orophilus* (Morelet, 1860).

### Bulimulus (Naesiotus) amastroides

Ancey, 1887

http://species-id.net/wiki/Bulimulus_(Naesiotus)_amastroides

Figs 14B, 14vi

Bulimulus (Naesiotus) amastroides Ancey 1887: 293; Wood and Gallichan 2008: 23.

#### Type locality.

[Ecuador] “Îles Galapagos”.

#### Label.

“I. Galapagos”, in Ancey’s handwriting.

#### Dimensions.

“Long., 9 1/2; diam., 4 2/3 mill.”; figured specimen H 9.25, D 4.5, W 7.0.

#### Type material.

RBINS/MT1866, lectotype (**design. n.**), ex Géret ex Ancey (Dautzenberg coll.).

#### Remarks.

Ancey writes that this species may only be compared to *Naesiotus calvus* (Sowerby, 1833), which was described from Isla San Salvador. However, Parent and Crispi (2006) found *Naesiotus amastroides* on Isla San Cristobal and suggested that its closest relative is *Naesiotus snodgrassi* (Dall, 1900).

#### Current systematic position.

Bulimulidae, *Naesiotus amastroides* (Ancey, 1887).

### Bulimulus (Rhinus) argentinus

Ancey, 1901

http://species-id.net/wiki/Bulimulus_(Rhinus)_argentinus

Figs 16A–B, 16i

Bulimulus (Rhinus) argentinus Ancey 1901: 92; Wood and Gallichan 2008: 25.
Rhinus argentinus  (Ancey); Breure 1978: 230, pl. 11 fig. 12; Breure 1979: 131.

#### Type locality.

“Gualeguaychu, province d’Entrerios, République Argentine”.

#### Label.

“Gualeguaychu / Prov. d’Entrerios / (Rép. Argentine)”, marked “types” in Ancey’s handwriting.

#### Dimensions.

“Long. 19–21, diam. 12 1/2–14 mill.”; figured specimen H 19.7, D 13.1, W 6.3.

#### Type material.

RBINS/MT1867, three syntypes, ex Géret ex Ancey (Dautzenberg coll.).

#### Current systematic position.

Simpulopsidae, *Rhinus argentinus* (Ancey, 1901).

### Placostylus (caledonicus) auriculata

Dautzenberg and Bouge in Dautzenberg, 1923

http://species-id.net/wiki/Placostylus_caledonicus_auriculata

Figs 9B, 9ii

Placostylus caledonicus  var. *auriculata* Dautzenberg and Bouge in Dautzenberg 1923: 148; Neubert et al. 2009: 104.

#### Type locality.

[New Caledonia] “Forêt du Mt. Ignambi, au dessus d’Oubatche 500 m. altit.”.

#### Label.

“N. Cal.” in Dautzenberg’s handwriting.

#### Dimensions.

Not given. Figured specimen H 81.7, D 42.0, W 5.7.

#### Type material.

RBINS/MT2339. One possible syntype (Dautzenberg coll.).

#### Remarks.

The specimen has a label glued onto the dorsal side “Bulimus / P[seu?]do-Caledonicus”. Dautzenberg (1923) explicitly stated there were two lots each with one specimen of this variety. This lot possibly may be attributed to one of these, although no specific locality is present with the specimen. Another lot, consisting of three specimens, is not considered type material. This variety has been synonymized with the nominate taxon by Neubert et al. (2009).

#### Current systematic position.

Bothriembryontidae, *Placostylus caledonicus* (Petit, 1845).

### Peronaeus baeri

Dautzenberg, 1901

http://species-id.net/wiki/Peronaeus_baeri

Figs 11C, 11iii

Peronaeus baeri Dautzenberg 1901a: 131; Dautzenberg 1901b: 214, pl. 8 figs 3–4; Fischer-Piette 1950: 169; Breure 1975b: 1140.
Bostryx baeri  (Dautzenberg); Breure 1979: 51.

#### Type locality.

“Iocos (Peruvia) Baer legit. 1900”; see remarks.

#### Label.

“Iocos Pérou / Baer legit.”, in Dautzenberg’s handwriting.

#### Dimensions.

“Altit. 14 1/2, latit. 3 1/2 millim.”; figured specimen H 13.6, D 3.1, W 10.1.

#### Type material.

RBINS/MT2336, three paralectotypes, Baer leg. (Dautzenberg coll.).

#### Remarks.

Dautzenberg (1901a) states in his paper that his description is based on four specimens. The holotype designation of a specimen in the MNHN collection (Fischer-Piette 1950) has to be interpreted as lectotype designation (Art. 74.6 ICZN); the measurements of this specimen closely match those given by Dautzenberg (Breure 1975b). The type locality is probably Jocos in Dept. La Libertad [8°14'S, 77°28'W].

#### Current systematic position.

Bulimulidae, *Bostryx baeri* (Dautzenberg, 1901).

### Bulimulus eschariferus bizonalis

Ancey, 1887

http://species-id.net/wiki/Bulimulus_eschariferus_bizonalis

Figs 14C, 14v

Bulimulus eschariferus  var. *bizonalis*Ancey 1887: 295; Wood and Gallichan 2008: 29.

#### Type locality.

[Ecuador] “îles Galapagos”.

#### Label.

“I Galapagos”, also stating “type de var. bizonalis Anc.”, in Ancey’s handwriting.

#### Dimensions.

Not given. Figured specimen H 16.0, D 6.3, W 7.5.

#### Type material.

RBINS/MT2337, one syntype, ex Géret ex Ancey ex Deshayes (Dautzenberg coll.).

#### Remarks.

Ancey did not state on how many specimens his description was based. Therefore, the specimen is considered a syntype.

#### Current systematic position.

Bulimulidae, *Naesiotus eschariferus* (Sowerby, 1833).

### Bulimulus blanfordianus

Ancey, 1903

http://species-id.net/wiki/Bulimulus_blanfordianus

Figs 4C–D, 4ii

Bulimulus blanfordianus Ancey 1903: 90; Wood and Gallichan 2008: 29.

#### Type locality.

“Iquico, Bolivia, 3500 m. above the sea (*fide* Fulton)”; see remarks.

#### Label.

“Iquico / Bolivia / 3500 mtr.”, in Fulton’s handwriting. Taxon label in Dautzenberg’s handwriting.

#### Dimensions.

“Long. 55, lat. 25 1/2 mill.”; figured specimen H 52.5, D 25.1, W 6.1.

#### Type material.

RBINS/MT1865, lectotype (**design. n.**), ex Géret ex Ancey (Dautzenberg coll.).

#### Remarks.

Ancey did not state on how many specimens his description was based. Despite the fact that the specimen is slightly smaller than published by Ancey, there is enough evidence to consider this shell as from the original type series. It is now designated lectotype.

The type locality probably refers to Dept. La Paz, Ikiko [16°34'S, 67°44'W], where elevations around 3500 m are found.

#### Current systematic position.

Megaspiridae, *Thaumastus (Thaumastus) blanfordianus* (Ancey, 1903).

### Bulimulus bonneti

Ancey, 1902

http://species-id.net/wiki/Bulimulus_bonneti

Figs 11A, 11i

Bulimulus bonneti Ancey 1902: 40, fig. 1; Fischer-Piette 1950: 170; Wood and Gallichan 2008: 29.
Peronaeus (Lissoacme?) bonneti  (Ancey); Breure 1975b: 1141.
Bostryx bonneti  (Ancey); Köhler 2007: 131, fig. 22.

#### Type locality.

“Bolivie (*teste* A. Bonnet)”.

#### Label.

“Bolivie”; see remarks.

#### Dimensions.

“Long. 23 1/2, lat. 13 mill.”; figured specimen H 21.9, D 14.7, W 5+.

#### Type material.

RBINS/MT2338, one paralectotype, ex Géret ex Ancey (Dautzenberg coll.).

#### Remarks.

Wood and Gallichan (2008) discussed the different labels of Fulton, Ancey and Dautzenberg. They argued for a syntypic status of this specimen, and for the material that is in the MNHN collection, which was regarded as the holotype by Fischer-Piette (1950); according to Art. 74.6 ICZN the MNHN specimen has to be regarded as a lectotype. The shell height of the MNHN-specimen is 22.0 mm, thus not corresponding to the original measurements given by Ancey. Breure (1975b) followed the designation as holotype by Fischer-Piette, being unaware of a second specimen in RBINS. The top of this specimen is damaged and it is likely that the original shell height was closer to Ancey’s dimensions.

#### Current systematic position.

Bulimulidae, *Bostryx bonneti* (Ancey, 1902).

### Bulimulus borellii

Ancey, 1897

http://species-id.net/wiki/Bulimulus_borellii

Figs 10F–G, 10iv

Bulimulus borellii Ancey 1897: 13; Wood and Gallichan 2008: 30.

#### Type locality.

[Bolivia, Dept. Tarija] “Mission de San Francisco, sur le haut-Pilcomayo en Bolivie”.

#### Label.

“Mission / de San Francisco / Haut-Pilcomayo, Bolivie”, in Ancey’s handwriting.

#### Dimensions.

[Measurements of three specimens given] “a) Long 34, lat. 11 mill.—b) Long 31, lat. 9 mill.—c) Long. 32, lat. 10 1/2 mill.”; largest figured specimen H 28.8, D 10.3, W 8.8.

#### Type material.

RBINS/MT2340, two syntypes, ex Géret ex Ancey, Borelli leg. (Dautzenberg coll.).

#### Remarks.

The specimens are smaller than the measurements given by Ancey, although they are from the type locality. This taxon was hitherto classified as *Drymaeus*. However, the sculpture of the protoconch shows very fine spiral lines and dispersed granules, more or less axially arranged. It thus belongs to *Bostryx* *(s.l.)*.

#### Current systematic position.

Bulimulidae, *Bostryx borellii* (Ancey, 1897) (**comb. n.**).

### Bulimulus (Drymaeus) carandaitiensis

Preston, 1907

http://species-id.net/wiki/Bulimulus_(Drymaeus)_carandaitiensis

Figs 10H, 10v

Bulimulus (Drymaeus) carandaitiensis Preston 1907: 491, fig. 4.
Drymaeus (Drymaeus) carandaitiensis  (Preston); Köhler 2007: 143, fig. 83.

#### Type locality.

[Bolivia, Dept. Chuquisaca] “Carandaiti, province of Cordillera, Bolivia, 1000 metres”.

#### Label.

“Carandaiti / Prov. of Cordillera 1000 metres / Bolivia (co-type)”, in Preston’s handwriting.

#### Dimensions.

“Alt. 35, diam. maj. 14 mill.”; figured specimen H 32.1, D 12.9, W 9.3.

#### Type material.

RBINS/MT2341, one syntype, ex Preston (Dautzenberg coll.).

#### Remarks.

This taxon was hitherto regarded a *Drymaeus* species, but the protoconch is smooth (not worn) in both specimens examined. It is here tentatively placed in *Bostryx*, despite the fact that the colour pattern of the specimen in RBINS is unlike the ones normally found in this genus.

#### Current systematic position.

Bulimulidae, *Bostryx carandaitiensis* (Preston, 1907) (**comb. n.**).

### Bulimulus montivagus chacoensis

Ancey, 1897

http://species-id.net/wiki/Bulimulus_montivagus_chacoensis

Figs 14D–E, 14i

Bulimulus montivagus  var. *chacoensis*Ancey 1897: 16; Wood and Gallichan 2008: 35.

#### Type locality.

[Bolivia, Dept. Tarija] “Caiza, Gran Chaco (Bolivie)”.

#### Label.

“Caiza, Chaco de Bolivie”, in Ancey’s handwriting.

#### Dimensions.

[Measurements of three specimens given] “a) Long. 22, lat. 7 1/2 mill—b) Long. 22, lat. 8 mill.—c) Long. 18, lat. 7 1/2 mill.”; figured specimen H 20.9, D 7.7, W 8.5.

#### Type material.

RBINS/MT2342, lectotype (**design. n.**), ex Géret ex Ancey, Borelli leg. (Dautzenberg coll.).

#### Remarks.

The RBINS material corresponds with the original measurements and is here designated lectotype. The shell shape, especially the flaring basal lip, and the number of whorls (8+) are not typical for *Naesiotus*, and this species is only tentatively placed here. Miquel (1989: 62) suggested a possible synonymy with *Naesiotus rocayanus* (d’Orbigny, 1835); this has to be ascertained by further comparison of type material.

#### Current systematic position.

Bulimulidae, *Naesiotus montivagus* (d’Orbigny, 1835).

### Bulimulus (Drymaeus) chacoensis

Preston, 1907

http://species-id.net/wiki/Bulimulus_(Drymaeus)_chacoensis

Figs 10E, 10iii

Bulimulus (Drymaeus) chacoensis Preston 1907: 491, fig. 5.
Bostryx chacoensis  (Preston); Köhler 2007: 132, fig. 23.

#### Type locality.

“To the north of the Rio Pilcomayo, Chaco, Bolivia”.

#### Label.

“N of riv. Pilcomayo / Chaco Bolivie 600 m / alt. (co-type)”; label in Dautzenberg’s handwriting, see remarks.

#### Dimensions.

“Alt. 30, diam. maj. 9.5 mm”; figured specimen H 30.1, D 10.1, W 8.1.

#### Type material.

RBINS/MT2343, one syntype, ex Preston (Dautzenberg coll.).

#### Remarks.

The specimen is not accompanied by an original Preston label. As Dautzenberg always accurately documented on his labels the source and date of his acquisition (in this case “Preston 14.xi.07”), there is hardly any doubt that this is an original type specimen. The protoconch is smooth, confirming that this taxon should be classified within *Bostryx* (cf. Breure 1979: 52).

#### Current systematic position.

Bulimulidae, *Bostryx chacoensis* (Preston, 1907).

### Otostomus colimensis

Rolle, 1895

http://species-id.net/wiki/Otostomus_colimensis

Figs 12C, 12iii

Otostomus colimensis Rolle 1895: 130.
Drymaeus (Drymaeus) colimensis  (Rolle); Breure 1979: 108; Köhler 2007: 144, fig. 89.

#### Type locality.

[Mexico] “Colima”.

#### Label.

“Colima Mexico”; label in Dautzenberg’s handwriting, see remarks.

#### Dimensions.

“Alt. 31, diam. 15 mm.”; figured specimen H 29.1, D 14.1, W 6.3.

#### Type material.

RBINS/MT2344, two paralectotypes, ex Rolle (Dautzenberg coll.).

#### Remarks.

This material is not accompanied by an original Rolle label, but Dautzenberg documented that he received the shells from him on 16.vii.1907. The fact that Rolle was a dealer may account for the delay between the time of publication and the acquisitions by Dautzenberg. The material is from the type locality and is here considered as syntypes. Köhler (2007) selected a lectotype from the ZMB material, thus these specimens are paralectotypes.

#### Current systematic position.

Bulimulidae, *Drymaeus (Drymaeus) colimensis* (Rolle, 1904).

### Bulimus coloratus

Nyst, 1845

http://species-id.net/wiki/Bulimus_coloratus

Figs 3A–B, 3i

Bulimus coloratus Nyst 1845a: 228, pl. fig. 2.

#### Type locality.

“la province de Cumana, dans la Colombie [sic, Venezuela]”.

#### Label.

“Colombie / Cumana”, indicating “Type (Nyst)” in Nyst’s handwriting.

#### Dimensions.

“49 millimètres de longueur sur 30 de largeur [H 49 D 30]”; lectotype H 47.3, D 29.2, W 4.7.

#### Type material.

RBINS/MT2345, lectotype (**design. n.**); MT2346, paralectotype, ex Nyst.

#### Remarks.

The type material was found in the RBINS collection and is now figured for the first time since the original publication. Of the two specimens present, one shows the ‘yellow shadow’ which is characteristic for this species, and is here designated lectotype. The taxon is only known from confirmed localities in northern Colombia; the original locality, which is in Venezuela, Edo. Sucre, seems erroneous. The type locality is now restricted to Sierra Nevada de Santa Marta.

#### Current systematic position.

Amphibulimidae, *Plekocheilus (Eurytus) coloratus* (Nyst, 1845).

### Bulimulus (Naesiotus) cucullinus

Dall, 1917

http://species-id.net/wiki/Bulimulus_(Naesiotus)_cucullinus

Figs 14A, 14ii

Bulimulus (Naesiotus) cucullinus Dall 1917: 377; Dall and Ochsner 1928: 166, pl. 8 figs 5–6; Boss et al. 1968: 96.
Naesiotus cucullinus  (Dall); Breure 1979: 68.

#### Type locality.

[Ecuador, Galápagos, Isla Española] “Hood Island, between 200 and 600 feet [61–183 m]”.

#### Label.

“Hood Is. 380 ft. / under stones, Galapagos” on a label of Stanford University.

#### Dimensions.

“Length of shell 19, diameter 9.5 mm.”; figured specimen H 17.7, D 8.6, W 6.8.

#### Type material.

RBINS/MT1833, two paratypes, Ochsner leg.

#### Remarks.

The material was donated by H.G. Schenk.

#### Current systematic position.

Bulimulidae, *Naesiotus cucullinus* (Dall, 1917).

### Plecochilus dalmasi

Dautzenberg, 1900

http://species-id.net/wiki/Plecochilus_dalmasi

Figs 3C–E, 3ii

Plecochilus  [sic, *Plekocheilus*] *dalmasi*Dautzenberg 1900: 151, pl. 9 fig. 1.

#### Type locality.

Not given.

#### Label.

[Colombia] “Sierra de Santa Marta”, in Dautzenberg’s handwriting.

#### Dimensions.

“Longit.: 26 millim., latit.: 16 millim.”; H 26.3, D 15.3, W 4.0.

#### Type material.

RBINS/MT668, lectotype (**design. n.**); MT2347, two paralectotypes (Dautzenberg coll.).

#### Remarks.

Dautzenberg did not state on how many specimens his description was based, but one of the specimens corresponds to the original dimensions and agrees with his figure. This shell is here designated lectotype. The label states that it was collected on 15.iii.1896, which corresponds with the date of excursion to Sierra de Santa Marta mentioned in the station list (Dautzenberg 1900: 147).

#### Current systematic position.

Amphibulimidae, *Plekocheilus (Eurytus) dalmasi* (Dautzenberg, 1900).

### Bulimulus (Protoglyptus) dejectus

Fulton, 1907

http://species-id.net/wiki/Bulimulus_(Protoglyptus)_dejectus

Figs 15B, 15iii

Bulimulus (Protoglyptus) dejectus Fulton 1907: 153, pl. 10 fig. 1.

#### Type locality.

[Brazil] “Santa Catharina (*fide* Linnaea Institute label)”.

#### Label.

“St. Catharina”; see remarks.

#### Dimensions.

“Maj. diam. 10, alt. 29 mm.”; figured specimen H 28.0, D 10.0, W 7.6.

#### Type material.

RBINS/MT2348, one syntype, ex Sowerby and Fulton (Dautzenberg coll.).

#### Remarks.

During a recent visit to the NHM, another specimen was found which will be designated lectotype (Breure and Ablett, unpublished data). The Brussels specimen thus will become a paralectotype. Although the specimen is not accompanied by an original Fulton label, Dautzenberg has noted on his label that he purchased this specimen from Sowerby and Fulton on 27.ii.1907. The generic placement of this taxon is somewhat puzzling. Three taxa may be considered, viz. *Protoglyptus*, *Naesiotus*, and *Rhinus*. The protoconch sculpture consists of axial wrinkles, partly broken into granules, which is a sculpture not characteristic for *Protoglyptus* nor *Naesiotus*. However, there is considerable variation in protoconch sculpture within these two taxa (Breure and Coppois 1978: Table II), and therefore it is difficult to decide on a generic placement on the basis of this characteristic alone. Further differences between the two genera are discussed by Breure and Coppois (1978: 163–165), who synonymized *Protoglyptus* with *Naesiotus*. The surface of the shell is partially sculptured with spiral series of granules, denoting an epidermis covered with hairs when fresh; this characteristic has been observed in all three groups. However, it must be noted that the shell shape is aberrant for *Rhinus*, and the anatomy of this species is unknown. Together with the results of Breure and Romero (in preparation), it seems justified to retain a tentative classification with *Protoglyptus*, which is now treated as a separate genus again. Study of live-collected specimens may shed new light on its classification.

#### Current systematic position.

Bulimulidae, *Protoglyptus dejectus* (Fulton, 1907).

### Placostylus porphyrostomus elata

Dautzenberg, 1923

http://species-id.net/wiki/Placostylus_porphyrostomus_elata

Figs 9A, 9i

Placostylus porphyrostomus elata Dautzenberg 1923: 148; Neubert et al. (2009): 79, 80.

#### Type locality.

“Nouvelle-Calédonie, St. Vincent (Coll. D. ex Rossiter)”.

#### Label.

“St. Vincent”, in Dautzenberg’s handwriting.

#### Dimensions.

“hauteur 88 millim. Diam. max. 36 millim.”; lectotype H 86.3, D 37.8, W 7.3.

#### Type material.

RBINS/MT702, lectotype (**design. n.**), Rossiter leg. (Dautzenberg coll.).

#### Remarks.

The specimen was marked by Dautzenberg as “type”. As he did not state how many specimens he had seen, the shell is here designated lectotype. Neubert et al. (2009) have placed this taxon in the synonymy of the nominate form of *Placostylus porphyrostomus* (Pfeiffer, 1851).

#### Current systematic position.

Bothriembryontidae, *Placostylus porphyrostomus* (Pfeiffer, 1851).

### Bulimus emaciatus

Morelet, 1863

http://species-id.net/wiki/Bulimus_emaciatus

Figs 11B, 11ii

Bulimus emaciatus Morelet 1863: 201, pl. 11 fig. 10.
Bostryx emaciatus  (Morelet); Breure 1978: 74, fig. 101 (lectotype designation); Breure 1979: 53.

#### Type locality.

[Peru] “dans les vallées et sur les plateaux de l’interieur de la Sierra, depuis Ayacucho jusqu’au Cuzco”.

#### Label.

“Pérou”. Locality and taxon label in Morelet’s handwriting.

#### Dimensions.

“Longit. 22; diam. 5 1/2 mill.”; figured specimen H 18.5, D 5.4, W 9.2.

#### Type material.

RBINS/MT2349, two syntypes, ex Morelet (Dautzenberg coll.).

#### Remarks.

Dautzenberg documented that these specimens originate from the Morelet collection. It is not clear if Dautzenberg had personal information from Morelet, when he added “Ayacucho” to his own label. Since a lectotype was designated by Breure (1978), the RBINS material will become paralectotypes.

#### Current systematic position.

Bulimulidae, *Bostryx emaciatus* (Morelet, 1863).

### Bulimulus ephippium

Ancey, 1904

http://species-id.net/wiki/_Bulimulus_ephippium

Figs 16C, 16ii

Bulimulus ephippium Ancey 1904: 102; Breure 1979: 62; Simone 2006: 118, fig. 361; Wood and Gallichan 2008: 44.
?Bulimulus ephippium  Ancey; Breure 1978: 144, pl. 11 fig. 8.

#### Type locality.

“Bahia, Brazil (*teste* H. Fulton)”.

#### Label.

“Bahia” in Fulton’s handwriting; taxon name in Ancey’s handwriting.

#### Dimensions.

“Longit. 20, diam. 12 mill.”; lectotype H 19.5, D 12.5, W 5.5.

#### Type material.

RBINS/MT2350, lectotype (**design. n.**), ex Géret ex Ancey (Dautzenberg coll.).

#### Remarks.

Breure (1978) mentioned that he had found syntypes in both the NHM and RBINS collections and figured the species for the first time. He redescribed the species but noted that its classification remained doubtful. Nevertheless he arranged it under *Bulimulus* in his 1979 revision. Upon restudying both specimens, it is clear that the protoconch sculpture is quite different from the pattern normal for that genus; especially the thin spiral lines give a strong hint that it should be separated. For that reason it cannot be classified with *Rhinus* either, although it bears resemblance in shell shape to species of that genus. It is now considered as *Simpulopsis (Eudioptus)*, where it is the largest species. The RBINS specimen is here designated as lectotype.

#### Current systematic position.

Simpulopsidae, *Simpulopsis (Eudioptus) ephippium* (Ancey, 1904).

### Bulimus fulminans

Nyst, 1843

http://species-id.net/wiki/Bulimus_fulminans

Figs 6A–C, 6i

Bulimus fulminans Nyst 1843: 261, pl. 7 fig. 1.

#### Type locality.

“la Colombie [sic, Venezuela], dans la province de Cumana [Edo. Sucre]”.

#### Label.

“Venezula (sic) / Cumana”, in Nyst’s handwriting.

#### Dimensions.

“60 millimètres de longeur sur 28 de largeur”; lectotype H 59.2, D 32.4, W 4.8.

#### Type material.

RBINS/MT2351, lectotype (**design. n.**), ex Nyst.

#### Remarks.

The specimen corresponds to the original measurements given by Nyst, whose label is dated 1874 and indicated “type”. As there is no evidence that the description of Nyst was based on one specimen, it is here designated lectotype.

#### Current systematic position.

Amphibulimidae, *Plekocheilus (Plekocheilus) fulminans* (Nyst, 1843).

### Bulimus funckii

Nyst, 1843

http://species-id.net/wiki/Bulimus_funckii

Figs 5A–B, 5i

Bulimus funckii Nyst 1843: 262, pl. 7 fig. 2.

#### Type locality.

“la Colombie [sic, Venezuela], dans la province de Cumana [Edo. Sucre]”.

#### Label.

“Venezuela / Cumana”, in Nyst’s handwriting.

#### Dimensions.

“90 millimètres de longeur sur 40 de largeur ”; lectotype H 86.3, D 44.5, W 5.5.

#### Type material.

RBINS/MT2352, lectotype (**design. n.**), ex Nyst.

#### Remarks.

Nyst (1843) did not indicate that he only had an unique type at hand. In his label dated 1874, he indicated this specimen as “type”; it is now designated as lectotype.

#### Current systematic position.

Amphibulimidae, *Dryptus funckii* (Nyst, 1843).

### Bulimulus gilderoyi

Van Mol, 1972

http://species-id.net/wiki/Bulimulus_gilderoyi

Figs 14G, 14iii

Bulimulus gilderoyi Van Mol 1972: 2, fig. 1.
Naesiotus gilderoyi  (Van Mol); Breure 1979: 69.

#### Type locality.

[Ecuador] “Galápagos, Santa Cruz, à proximité du Cerro Coralon”.

#### Label.

“Galapagos, Santa Cruz, (...) Cerro Coralon”, in Van Mol’s handwriting.

#### Dimensions.

“Hauteur 25.3 Largeur max. 15.5 (mm)”; holotype H 25.4, D 15.6 W 6.6.

#### Type material.

RBINS/MT106, holotype; MT107, one paratype; MT108, 19 paratypes, all Van Mol leg., 27.x.1970.

#### Remarks.

This taxon was published on 10.ix.1972; A.G. Smith published a paper in which he described the same species as *Naesiotus cavagnaroi* on 21.i.1972. Breure and Coppois (1978: 170) synonymized *Bulimulus gilderoyi* Van Mol with *Naesiotus cavagnaroi* A.G. Smith, as a junior subjective synonym.

#### Current systematic position.

Bulimulidae, *Naesiotus cavagnaroi* A.G. Smith, 1972.

### Bulimulus alternatus hesperius

Pilsbry and Ferriss, 1924

http://species-id.net/wiki/Bulimulus_alternatus_hesperius

Figs 15C, 15iv

Bulimulus alternatus hesperius  Pilsbry and Ferriss in Ferriss 1924: 40; H.B. Baker 1962: 10.

#### Type locality.

[U.S.A., Texas] “east side of the Pecos [river] at the High Bridge”.

#### Label.

“near Pecos river / Texas”; see remarks.

#### Dimensions.

“34 mm. long, 17.4 mm. diameter”; figured specimen H 34.2, D 17.2, W 7.7.

#### Type material.

RBINS/MT2353, two probable paratypes, ex Eyerdam, J.H. Ferriss leg., 1924 (Dautzenberg coll.).

#### Remarks.

Pilsbry and Ferriss (in Ferris 1924) state they have selected a specimen as type, which according to H.B. Baker (1962) is holotype ANSP 84627a. As the label states that the material was collected by Ferriss in 1924, it probably belongs to the original type series. The locality—although more vaguely formulated on the label—corresponds to the area mentioned by Ferriss (1924) for the type series.

#### Current systematic position.

Bulimulidae, *Rabdotus alternatus* (Say, 1829).

### Bulimulus (Ataxus) huayaboensis

Dautzenberg, 1901

http://species-id.net/wiki/Bulimulus_(Ataxus)_huayaboensis

Figs 11H, 11viii

Bulimulus (Ataxus) huayaboensis Dautzenberg 1901c: 311, pl. 9 figs 8–9.
Bulimulus huayaboensis  Dautzenberg; Fischer-Piette 1950: 170.
Bostryx (Ataxus) huayaboensis  (Dautzenberg); Breure 1975b: 1140.
Bostryx huayaboensis  (Dautzenberg); Breure 1978: 91, pl. 7 figs 16–17.

#### Type locality.

“Huayabo (Marañon) Pérou, à 2000 m. d’altitude”.

#### Label.

“Huayabo Marañon / Pérou 2000m alt.”, in Dautzenberg’s handwriting.

#### Dimensions.

“Long. 24, diam. maj. 10 millim.”; figured specimen H 22.9, D 8.5, W 7.2.

#### Type material.

RBINS/MT2354, five paralectotypes, Baer leg. (Dautzenberg coll.).

#### Remarks.

The holotype designation of a specimen in the MNHN collection (Fischer-Piette 1950) has to be interpreted as lectotype designation (Art. 74.6 ICZN); the measurements of this specimen closely match those given by Dautzenberg (Breure 1975b).

#### Current systematic position.

Bulimulidae, *Bostryx huyaboensis* (Dautzenberg, 1901).

### Bulimus poecilus icterica

Ancey, 1892

http://species-id.net/wiki/Bulimus_poecilus_icterica

Figs 12D, 12iv

Bulimus poecilus icterica Ancey 1892: 92; Wood and Gallichan 2008: 54.

#### Type locality.

“Province of Matto-Grosso, Brazil (Germain)”.

#### Label.

“Matto-Grosso”, in Ancey’s handwriting.

#### Dimensions.

“Long. 29, alt. (obl.) 13 mill.”; figured specimen 28.1, D 13.4, W 6.6.

#### Type material.

RBINS/MT1881, one syntype, ex Géret ex Ancey, P. Germain leg. (Dautzenberg coll.).

#### Remarks.

Ancey (1892) states that he had seen two specimens. The whereabouts of the other syntype is unknown.

#### Current systematic position.

Bulimulidae, *Drymaeus (Drymaeus) poecilus ictericus* (Ancey, 1892).

### Bulimulus (Drymaeus) interruptus

Preston, 1909

http://species-id.net/wiki/Bulimulus_(Drymaeus)_interruptus

Figs 12E, 12v

Bulimulus (Drymaeus) interruptus Preston 1909: 511, pl. 10 fig. 1.
Drymaeus (Mesembrinus) interruptus  (Preston); Breure 1979: 120; Köhler 2007: 151, fig. 119.

#### Type locality.

“Merida, Venezuela”.

#### Label.

“Merida Venezuela”; see remarks.

#### Dimensions.

“Alt. 25, diam. maj. 10.5 mm”; figured specimen H 23.6, D 10.6, W 6.1

#### Type material.

RBINS/MT2257, one syntype, ex Preston (Dautzenberg coll.).

#### Remarks.

The specimen is not accompanied by the original Preston label, but Dautzenberg documented that the shell was acquired on 19.xii.1907 from Preston as a “co-type”. It is therefore considered a syntype. See also *pallidus* Preston, 1909.

#### Current systematic position.

Bulimulidae, *Drymaeus (Mesembrinus) granadensis* (Pfeiffer, 1848).

### Peronaeus (Peronaeus) iocosensis

Dautzenberg, 1901

http://species-id.net/wiki/Bulimulus_(Drymaeus)_interruptus

Figs 11D, 11iv

Peronaeus iocosensis Dautzenberg 1901a: 131; Dautzenberg 1901b: 213, pl. 8 figs 1–2; Fischer-Piette 1950: 169.
Peronaeus (Peronaeus) iocosensis  Dautzenberg; Breure 1975b: 1141.
Bostryx iocosensis  (Dautzenberg); Breure 1979: 55.

#### Type locality.

“Iocos (Peruvia) Baer legit. 1900”; see remarks.

#### Label.

“Iocos Pérou / Baer legit.”, in Dautzenberg’s handwriting.

#### Dimensions.

“Altit. 13, latit. 3 millim.”; figured specimen H 11.3, D 2.8, W 10.3.

#### Type material.

RBINS/MT2355, two paralectotypes, Bear leg., 1900 (Dautzenberg coll.).

#### Remarks.

Dautzenberg (1901a) remarked that he had seen three specimens. The holotype designation of a specimen in the MNHN collection (Fischer-Piette 1950) has to be interpreted as lectotype designation (Art. 74.6 ICZN); the measurements of this specimen closely match those given by Dautzenberg (Breure 1975b). The RBINS material should thus be considered paralectotypes. The type locality is probably Jocos in Dept. La Libertad [8°14'S, 77°28'W].

#### Current systematic position.

Bulimulidae, *Bostryx iocosensis* (Dautzenberg, 1901).

### Drymaeus jousseaumei

Dautzenberg, 1901

http://species-id.net/wiki/Drymaeus_jousseaumei

Figs 13A, 13i

Drymaeus jousseaumei Dautzenberg 1901c: 308; Fischer-Piette 1950: 170; Breure 1975b: 1151;
Drymaeus (Drymaeus) jousseaumei  Dautzenberg; Breure 1979: 110.

#### Type locality.

“Rio Mixiollo, Province Huallaga, Pérou” [Dept. San Martin].

#### Label.

“Rio Mixiolla / prov. Huallaga Pérou”, in Dautzenberg’s handwriting.

#### Dimensions.

“Long. 50, diam. maj. 22 mill.”; figured specimen H 47.6, D 21.6, W 6.9.

#### Type material.

RBINS/MT2356, two paralectotypes, Baer leg. (Dautzenberg coll.).

#### Remarks.

Dautzenberg (1901c: 309) reports to have seen three specimens. The holotype designation of a specimen in the MNHN collection (Fischer-Piette 1950) has to be interpreted as lectotype designation (Art. 74.6 ICZN); the measurements of this specimen closely match those given by Dautzenberg (Breure 1975b). The RBINS material should thus be considered paralectotypes.

#### Current systematic position.

Bulimulidae, *Drymaeus (Drymaeus) jousseaumei* Dautzenberg, 1901.

### Thaumastus juana

Cousin, 1887

http://species-id.net/wiki/Thaumastus_juana

Figs 10C–D, 10ii

Thaumastus juana Cousin 1887: 228, pl. 4 fig. 10.
Peronaeus (Lissoacme) juana  (Cousin); Breure 1975: 1141, pl. 6 fig. 5.

#### Type locality.

[Ecuador, Prov. Azuay] “Gualacco [sic, Gualaceo], province de Cuença”.

#### Label.

“chemin à Gualacco, rives du Paute, avant le pont”, in Cousin’s handwriting.

#### Dimensions.

“long., 20 à 23 mm; diam. 10 à 12 mm”; figured specimen H 19.1, D 8.76, W 7.7.

#### Type material.

RBINS/MT2357, seven paralectotypes, Cousin leg. (Dautzenberg coll.).

#### Remarks.

The lectotype is in the MNHN collection (Breure 1975b). According to the inventory of Cousin’s collection, there were originally 35 specimens present.

#### Current systematic position.

Bulimulidae, *Bostryx juana* (Cousin, 1887).

### Orphnus thompsoni lutea

Cousin, 1887

http://species-id.net/wiki/Orphnus_thompsoni_lutea

Figs 7A, 7i

Orphnus thompsoni  var. *lutea*Cousin 1887: 212.

#### Type locality.

[Ecuador, Prov. Azuay] “Cuença”.

#### Label.

“Cuenca Ecuador”, label in Dautzenberg’s handwriting.

#### Dimensions.

Not given. Lectotype H 77.6, D 37.2, W 6.7.

#### Type material.

RBINS/MT2358, lectotype (**design. n.**); MT2359, five paralectotypes, ex Cousin (Dautzenberg coll.).

#### Remarks.

In the inventory of Cousin’s collection, a total of 11 specimens is mentioned for this variety. From among the specimens in RBINS, a lectotype is here designated and the taxon is now considered a junior subjective synonym of *Bulimus thompsonii* Pfeiffer, 1845 (**syn. n.**).

#### Current systematic position.

Orthalicidae, *Kara thompsonii* (Pfeiffer, 1845) (**comb. n.**).

### Bulimulus (Naesiotus) lycodus

Dall, 1917

http://species-id.net/wiki/Bulimulus_(Naesiotus)_lycodus

Figs 14F, 14iv

Bulimulus (Naesiotus) lycodus Dall 1917: 379; Dall and Ochsner 1928: 168, pl. 8 figs 11, 15–16; Boss et al. 1968: 193.
Naesiotus lycodus  (Dall); Breure 1979: 70.

#### Type locality.

[Ecuador, Galápagos, Isla Santa Cruz] “Indefatigable Island at 450 to 550 feet elevation [137–167 m]”.

#### Label.

“Indefatigable Island” on label of Stanford University.

#### Dimensions.

“Length of shell 11, diameter 8 mm.”; figured specimen H 11.4, D 7.1, W 6.2.

#### Type material.

RBINS/MT1834, one paratype, Ochsner leg.

#### Remarks.

The material is accompanied by a label from Stanford University and was donated by H.G. Schenck.

#### Current systematic position.

Bulimulidae, *Naesiotus lycodus* (Dall, 1917).

### Bulimulus mazei

Crosse, 1874

http://species-id.net/wiki/Bulimulus_mazei

Figs 15A, 15ii

Bulimulus mazei Crosse 1874a: 118; Crosse 1874b: 202, pl. 4 fig. 3; Fischer-Piette 1950: 80.
Naesiotus mazei  (Crosse); Breure 1975a: 84, pl. 8 fig. 11; Breure 1975b: 1146; Breure 1979: 70.

#### Type locality.

[Martinique] “Massif des Pitons, ad 730 met. altitudinem supra mare (Mazé)”.

#### Label.

“Martinique”, “Massif des Pitons”, in handwriting of Crosse.

#### Dimensions.

“Long. 19 mill., diam. maj. 7”; figured specimen H 14.5, D 5.9, W 6.4.

#### Type material.

RBINS/MT2360, one paralectotype, ex Crosse, Mazé leg. (Dautzenberg coll.).

#### Remarks.

The holotype designation of a specimen in the MNHN collection (Fischer-Piette 1950) has to be interpreted as lectotype designation (Art. 74.6 ICZN); the measurements of this specimen closely match those given by Crosse (Breure 1975b). The RBINS material should thus be considered a paralectotype; it is a subadult specimen.

#### Current systematic position.

Bulimulidae, *Protoglyptus mazei* (Crosse, 1874) (**comb. n.**).

### Bulimus melanocheilus

Nyst, 1845

http://species-id.net/wiki/Bulimus_melanocheilus

Figs 4A–B, 4i

Bulimus melanocheilus Nyst 1845b: 149, pl. 2 fig. 3.

#### Type locality.

“l’Amérique mériodionale, au Pampas”.

#### Label.

“Amerique mériodionale / (Pampas) / Pampaijacu”, in Nyst’s handwriting.

#### Dimensions.

“longeur de 80 millimètres sur 35 de largeur”; lectotype H 78.5, D 36.6, W 6.2.

#### Type material.

RBINS/MT2361, lectotype (**design. n.**), ex Nyst.

#### Remarks.

The specimen is accompanied by a label of Nyst, dated 1874, and marked “type Nyst”. Nyst (1845b) writes “cette belle coquille”, but this is here—following ICZN Recommendation 73F—not interpreted as a holotype; the specimen is now designated as lectotype. The locality “ Pampaijacu” (or Pampayacu) is found only in Peru, both in Dept. Lima and Dept. Huánuco. Since the former is at 3800 m and beyond the altitudinal range known for this genus (0–2300 m; Breure 1979), the type locality is probably Pampayacu in Dept. Huánuco [09°33'S, 75°54'W, 1150 m].

#### Current systematic position.

Megaspiridae, *Thaumastus (Thaumastus) melanocheilus* (Nyst, 1845).

### Bostryx metagyra

Pilsbry and Olsson, 1949

http://species-id.net/wiki/Bostryx_metagyra

Bostryx metagyra Pilsbry and Olsson 1949: 9, fig. 12; H.B. Baker 1963: 229; Breure 1979: 56; Neubert and Janssen 2004: 217, pl. 7 fig. 83.

#### Type locality.

“Peru”.

#### Label.

“Peru”.

#### Type material.

RBINS/MT2362, seven paratypes.

#### Remarks.

The specimens were exchanged with FMNH and originate from the original series as deposited in the Museo de Historia Natural in Lima; see also Neubert and Janssen (2004) for a recent re-illustration.

#### Current systematic position.

Bulimulidae, *Bostryx metagyra* Pilsbry and Olsson, 1949.

### Bulimulus (Bostryx) moniezi

Dautzenberg, 1896

http://species-id.net/wiki/Bulimulus_(Bostryx)_moniezi

Figs 11G, 11vii

Bulimulus (Bostryx) moniezi Dautzenberg 1896: 224, pl. 7 fig. 3.
Bostryx moniezi  (Dautzenberg); Breure 1979: 56.

#### Type locality.

“le Haut-Pérou”.

#### Label.

“Andes / Pérou” (handwriting of Dupuis?).

#### Dimensions.

“Longit. 14 millim., latit. 6 1/2 millim.”; figured specimen H 13.9, D 6.2, W 9.4.

#### Type material.

RBINS/MT1830, one syntype, ex P. Dupuis.

#### Remarks.

The specimen is labelled “co-type” but fits the original measurements. It is not accompanied by a Dautzenberg label; however, its type status is not questioned here as Paul Dupuis is known to have been in close contact with Dautzenberg (Duchamps 1999).

#### Current systematic position.

Bulimulidae, *Bostryx moniezi* (Dautzenberg, 1896).

### Orphnus thompsoni nigricans

Cousin, 1887

http://species-id.net/wiki/Orphnus_thompsoni_nigricans

Figs 7B, 7ii

Orphnus thompsoni  var. *nigricans*Cousin 1887: 212.

#### Type locality.

[Ecuador, Prov. Azuay] “Cuença”.

#### Label.

“Cuenca, Ecuador”; see remarks.

#### Dimensions.

Not given. Lectotype H 62.8, D 30.3, W 6.1.

#### Type material.

RBINS/MT2363, lectotype (**design. n.**); MT2364, three paralectotypes, ex Cousin (Dautzenberg coll.).

#### Remarks.

The original Cousin label mentions the taxon name only; the locality has been added by Dautzenberg, probably on the basis of Cousin’s paper. One specimen was found with the Cousin label stuffed inside the aperture and is here designated lectotype. The taxon is considered a junior subjective synonym of *Bulimus thompsonii* Pfeiffer, 1845 (**syn. n.**).

#### Current systematic position.

Orthalicidae, *Kara thompsonii* (Pfeiffer, 1845) (**comb. n.**).

### Thaumastus nystianus nigricans

Cousin, 1887

http://species-id.net/wiki/Thaumastus_nystianus_nigricans

Figs 12B, 12ii

Thaumastus nystianus  var. *nigricans*Cousin 1887: 220.

#### Type locality.

[Ecuador, Prov. Pichincha] “les bords du chemin qui conduit de Pomasqui à Chilguiltina”.

#### Label.

“chemin de Pomasqui à Chilguiltina” in Dautzenberg’s handwriting; see remarks.

#### Dimensions.

Not given. Figured specimen H 37.3, D 17.7, W 6.3.

#### Type material.

RBINS/MT2365, 13 possible syntypes, ex Cousin (Dautzenberg coll.).

#### Remarks.

The material is not accompanied by an original Cousin label, but it originates from Cousin; the specimens are treated herein as possible syntypes. This taxon is now considered a junior subjective synonym of *Bulimus nystianus* Pfeiffer, 1853 (**syn. n.**).

#### Current systematic position.

Bulimulidae, *Drymaeus (Drymaeus) nystianus* (Pfeiffer, 1853).

### Orphnus thompsoni olivacea

Cousin, 1887

http://species-id.net/wiki/Orphnus_thompsoni_olivacea

Figs 7C, 7iii

Orphnus thompsoni  var. *olivacea*Cousin 1887: 212.

#### Type locality.

[Ecuador, Prov. Azuay] “Cuença”.

#### Label.

“Cuenca, Ecuador”; see remarks.

#### Dimensions.

Not given. Lectotype H 64.5, D 32.9, W 6.2.

#### Type material.

RBINS/MT2366, lectotype (**design. n.**); MT2367, three paralectotypes, ex Cousin (Dautzenberg coll.).

#### Remarks.

The original Cousin label mentions the taxon name only; the locality has been added by Dautzenberg, probably on the basis of Cousin’s paper. One specimen was found with the Cousin label stuffed inside the aperture and is here designated lectotype. The taxon is considered a junior subjective synonym of *Bulimus thompsonii* Pfeiffer, 1845 (**syn. n.**).

#### Current systematic position.

Orthalicidae, *Kara thompsonii* (Pfeiffer, 1845) (**comb. n.**).

### Bulimulus (Drymaeus) interruptus pallidus

Preston, 1909

http://species-id.net/wiki/Bulimulus_(Drymaeus)_interruptus_pallidus

Figs 12F, 12vi

Bulimulus (Drymaeus) interruptus  var. *pallidus*Preston 1909: 511, fig. 2.

#### Type locality.

“Merida, Venezuela”.

#### Label.

“Merida (Venezuela)”; see remarks.

#### Dimensions.

Not given. Figured specimen H 24.0, D 11.0, W 6.2.

#### Type material.

RBINS/MT2258, one syntype, ex Preston (Dautzenberg coll.).

#### Remarks.

The specimen is not accompanied by a Preston label, but Dautzenberg documented that he acquired the specimen from Preston on 19.xii.1907; the type status of the specimen is here not contested and it is regarded as a syntype.

#### Current systematic position.

Bulimulidae, *Drymaeus (Mesembrinus) granadensis* (Pfeiffer, 1848).

### Placostylus (Maoristylus) ambagiosus paraspiritus

Powell, 1951

http://species-id.net/wiki/Placostylus_(Maoristylus)_ambagiosus_paraspiritus

Figs 8B, 8ii

Placostylus (Maoristylus) ambagiosus paraspiritus Powell 1951: 137, pl. 28 fig. 7.

#### Type locality.

[New Zealand, North Island] “one mile south of Cape Maria van Diemen”.

#### Label.

“Headland, 1 mile S. of Cape Maria van / Diemen.”.

#### Dimensions.

“Height 72.25 mm. Diameter 32.0 mm.”; figured specimen H 82.2, D 39.2, W 5.8.

#### Type material.

RBINS/MT1969, one paratype, ex L. Germain (Dautzenberg coll.).

#### Remarks.

The specimen is labelled “paratypes”, despite the fact that it is only one specimen.

#### Current systematic position.

Bothriembryontidae, *Placostylus ambagiosus* (Suter, 1906).

### Bulimulus (Ataxus) perforatus

Haas, 1951

http://species-id.net/wiki/Bulimulus_(Ataxus)_perforatus

Bulimulus (Ataxus) perforatus Haas 1951: 518, fig. 106.
Bostryx perforatus  (Haas); Breure 1979: 56.

#### Type locality.

“Ninabamba on the Pampas River, an affluent of the Apurimac River, Peru. Altitude 2,000 m”.

#### Label.

“Ninabamba, Rio Pampas, 2.000 m. / alt., Peru” typewritten by Weyrauch.

#### Dimensions.

“Height 19.5 mm, width 8.2 mm.”; largest specimen H 20.4, D 7.53, W 9.4.

#### Type material.

RBINS/MT2368, two paratypes, W. Weyrauch leg.

#### Remarks.

The material was exchanged on the basis of material from the Dautzenberg collection (see Introduction) with FMNH, where the holotype of this taxon is kept.

#### Current systematic position.

Bulimulidae, *Bostryx perforatus* (Haas, 1951).

### Bulimulus pollonerae

Ancey, 1897

http://species-id.net/wiki/Bulimulus_pollonerae

Figs 14H–I, 14vii

Bulimulus pollonerae Ancey 1897: 17, fig. 10; Wood and Gallichan 2008: 77.

#### Type locality.

“San Lorenzo, province de Jujuy, République de Argentine”.

#### Label.

“San Lorenzo / prov. de Jujuy / Rep. Argentine”, in Ancey’s handwriting.

#### Dimensions.

“Long. 15 1/2, lat. 6 1/2 mm.”; lectotype H 15.3, D 6.33, W 7.7.

#### Type material.

RBINS/MT2369, lectotype (**design. n.**); MT2370, one paralectotype, ex Géret ex Ancey, Borelli leg. (Dautzenberg coll.).

#### Remarks.

The type material consists of a subadult and an adult specimen; the latter is here designated lectotype.

#### Current systematic position.

Bulimulidae, *Naesiotus pollonerae* (Ancey, 1897).

### Leucocharis porphyrochila

Dautzenberg and Bernier, 1901

http://species-id.net/wiki/Leucocharis_porphyrochila

Figs 8A, 8i

Leucocharis porphyrochila Dautzenberg and Bernier 1901: 215, pl. 7 figs 5–6; Fischer-Piette 1950: 170.

#### Type locality.

“Nouvelle-Calédonie”.

#### Label.

“Houaïlou N. Caléd.”, in Dautzenberg’s handwriting.

#### Dimensions.

“Altit. 43 millim., latit. 22 millim.”; figured specimen H 41.9, D 22.0, W 5.9.

#### Type material.

RBINS/MT2371, two syntypes, ex Bernier (Dautzenberg coll.).

#### Remarks.

Two additional syntypes are in the MNHN-collection (V. Héros, pers. commun.).

#### Current systematic position.

Bothriembryontidae, *Aspastus (Leucocharis) porphyrochila* (Dautzenberg and Bernier, 1901).

### Bulimulus (Scutalus) sanborni

Haas, 1947

http://species-id.net/wiki/Bulimulus_(Scutalus)_sanborni

Figs 15D, 15i

Bulimulus (Scutalus) sanborni Haas 1947: 176, fig. 33.
Scutalus (Vermiculatus) sanborni  (Haas); Breure 1979: 86.

#### Type locality.

“Carhuamayo, basin of Lake Junín, Department of Loreto [sic, Junín], 15,000–18,000 feet [4572–5486 m]”.

#### Label.

“Carhuamayo above Lake Junin / Dept. Loreto, Peru” typewritten (by FMNH technician?).

#### Dimensions.

“Height 10.6 mm, width 6 mm”; figured specimen H 10.0, D 5.5 W 4.5.

#### Type material.

RBINS/MT2372, three paratypes, C.C. Sanborn leg., 1946.

#### Remarks.

The material was acquired by exchange with FMNH on the basis of material from the Dautzenberg collection (see Introduction).

#### Current systematic position.

Bulimulidae, *Kuschelenia (Vermiculatus) sanborni* (Haas, 1947) (**comb. n.**).

### Drymaeus scoliodes

Dautzenberg, 1901

http://species-id.net/wiki/Drymaeus_scoliodes

Figs 13B, 13ii

Drymaeus scoliodes Dautzenberg 1901c: 309, pl. 9 figs 6–7; Fischer-Piette 1950: 170.
Drymaeus (Drymaeus) scoliodes  (Dautzenberg); Breure 1979: 114.
Cochlorina scoliodes  (Dautzenberg); Breure 1975b: 1149, pl. 2 fig. 5.

#### Type locality.

“Rio Mixiolla, province Huallaga, Pérou”.

#### Label.

“Rio Mixiolla prov. / Huallaga Pérou”, in Dautzenberg’s handwriting.

#### Dimensions.

“Long. 63, diam. maj. 22 millim.”; figured specimen H 40.7, D 21.3, W 6.8.

#### Type material.

RBINS/MT2377, one paralectotype, Baer leg. (Dautzenberg coll.).

#### Remarks.

The specimen is not marked as type, but corresponds to the data as given by Dautzenberg (1901). The holotype designation of a specimen in the MNHN collection (Fischer-Piette 1950) has to be interpreted as lectotype designation (Art. 74.6 ICZN); the measurements of this specimen closely match those given by Dautzenberg (Breure 1975b). The RBINS material should thus be considered a paralectotype as Dautzenberg states he had seen two specimens.

#### Current systematic position.

Bulimulidae, *Drymaeus (Drymaeus) scoliodes* (Dautzenberg, 1901).

### Bulimulus (Drymaeus) solidus

Preston, 1907

http://species-id.net/wiki/Bulimulus_(Drymaeus)_solidus

Figs 13C, 13iii

Bulimulus (Drymaeus) solidus Preston 1907: 494, fig. 9.
Drymaeus (Drymaeus) solidus  (Preston); Köhler 148, fig. 109.

#### Type locality.

“Bogota, United States of Colombia”.

#### Label.

“Bogota / U.S. Colombia”, in Preston’s handwriting.

#### Dimensions.

“Alt. 32.5, diam. maj. 15 mm.”; figured specimen H 33.8, D 17.3, W 6.2.

#### Type material.

RBINS/MT2259, one syntype, ex Preston (Dautzenberg coll.).

#### Remarks.

The specimen is marked “co-type” on the original label.

#### Current systematic position.

Bulimulidae, *Drymaeus (Drymaeus) solidus* (Preston, 1907).

### Bulimus spiculatus

Morelet, 1860

http://species-id.net/wiki/Bulimus_spiculatus

Figs 11E, 11v

Bulimus spiculatus Morelet 1860: 375.
Bostryx spiculatus  (Morelet); Breure 1978: 122; Breure 1979: 58;

#### Type locality.

No type locality given [interior of Peru].

#### Label.

“Pérou”; see remarks.

#### Dimensions.

“Longit. 20; diam. 4 mill.”; figured specimen H 21.8, D 4.26, W 11.4.

#### Type material.

RBINS/MT2373, four probable syntypes, ex Morelet (Dautzenberg coll.).

#### Remarks.

Dautzenberg documented that he received the specimens from the Morelet collection (“ex auctore”). The material is regarded as probable syntypes. It may be noted that Breure (1978: 122) designated a lectotype from among the material in NHM, while other type material is present in MHNG.

#### Current systematic position.

Bulimulidae, *Bostryx spiculatus* (Morelet, 1860).

### Bulimulus (Protoglyptus) subcostatus

Haas, 1948

http://species-id.net/wiki/Bulimulus_(Protoglyptus)_subcostatus

Bulimulus (Protoglyptus) subcostatus  Haas 1948: 190, fig. 39.
Naesiotus subcostatus  (Haas); Breure 1979: 72.

#### Type locality.

“Jaën, Department of Cajamarca, Peru. Altitude 1,500–2,100 feet [457–604 m]”.

#### Label.

“Jaen, Camarca [sic], Peru, 550–700 m / alt. W. Weyrauch leg.” typewritten (by FMNH technician?).

#### Dimensions.

“Height 12.4 mm, width 5 mm”.

#### Type material.

RBINS/MT2379, two paratypes, W. Weyrauch leg.

#### Remarks.

Haas (1948) did not mention how many paratypes he had in his material. It is thus possible that these specimens formed part of the original series, as they are labelled “paratypes”. The species was adequately figured by Haas. The material was received in exchange from FMNH where the holotype is kept.

#### Current systematic position.

Bulimulidae, *Naesiotus subcostatus* (Haas, 1948).

### Bulimus veruculum

Morelet, 1860

http://species-id.net/wiki/Bulimus_veruculum

Figs 11F, 11vi

Bulimus veruculum Morelet 1860: 376.

#### Type locality.

No type locality given [interior of Peru].

#### Label.

“Balsa de Cocharcas / Pérou”, see remarks.

#### Dimensions.

“Longit. 24; diam. 4 1/2 mill.”; figured specimen H 21.9, D 3.85, W 16.7.

#### Type material.

RBINS/MT2374, four syntypes, ex Morelet (Dautzenberg coll.).

#### Remarks.

Dautzenberg documented that he received the specimens from the Morelet collection. The material is regarded as syntypes.

#### Current systematic position.

Bulimulidae, *Bostryx veruculum* (Morelet, 1860).

### Placostylus (Maoristylus) whareana

Powell, 1951

http://species-id.net/wiki/Placostylus_(Maoristylus)_whareana

Figs 8C, 8iii

Placostylus (Maoristylus) ambagiosus whareana Powell 1951: 135, pl. 28 fig. 2.

#### Type locality.

“Whareana, east coast between Waikuku Beach and Parengarenga”.

#### Label.

“Whareana Valley, S. of North Cape”.

#### Dimensions.

“Height 79.5 mm. Diameter 35.0 mm.”; figured specimen H 65.9, D 30.7, W 7.0.

#### Type material.

RBINS/MT376, one paratype, ex L. Germain.

#### Remarks.

The label reads “paratypes”, but only one specimen was found.

#### Current systematic position.

Bothriembryontidae, *Placostylus ambagiosus* (Suter, 1906).

### Orphnus thompsoni zebra

Cousin, 1887

http://species-id.net/wiki/Orphnus_thompsoni_zebra

Figs 7D, 7iv

Orphnus thompsoni  var. *zebra*Cousin 1887: 212.

#### Type locality.

[Ecuador, Prov. Cañar] “près Azagues [sic, Azogues], sur la pente, à environs 2400m alt.”.

#### Label.

“près Azagues sur la / pente à envir. 2400m alt.”; see remarks.

#### Dimensions.

Not given. Lectotype H 46.4, D 25.4, W 5.4.

#### Type material.

RBINS/MT2375, lectotype (**design. n.**); MT2376, nine paralectotypes, ex Cousin (Dautzenberg coll.).

#### Remarks.

The original Cousin label mentions the taxon name only; the locality has been added by Dautzenberg, probably on the basis of Cousin’s paper. Several specimens are juvenile or damaged. One specimen is here designated lectotype. The taxon is considered a junior subjective synonym of *Kara thompsonii* (Pfeiffer, 1851) (**syn. n.**).

#### Current systematic position.

Orthalicidae, *Kara thompsonii* (Pfeiffer, 1851) (**comb. n.**).

## Plates 3 to 16

**Figure 3.**
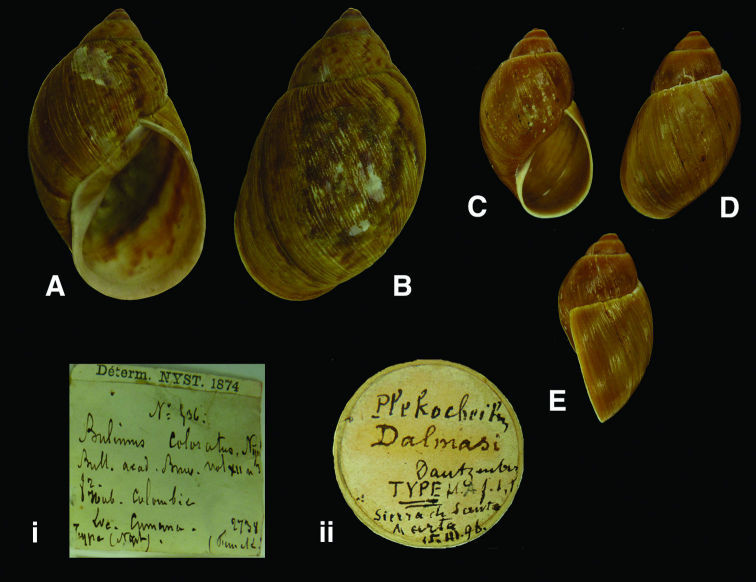
**A–B, i** *Plekocheilus (Eurytus) coloratus* (Nyst, 1845), lectotype RBINS/MT2345 (H=47.3). **C–E, ii** *Plekocheilus (Eurytus) dalmasi* (Dautzenberg, 1900), lectotype RBINS/MT668 (H=26.3).

**Figure 4.**
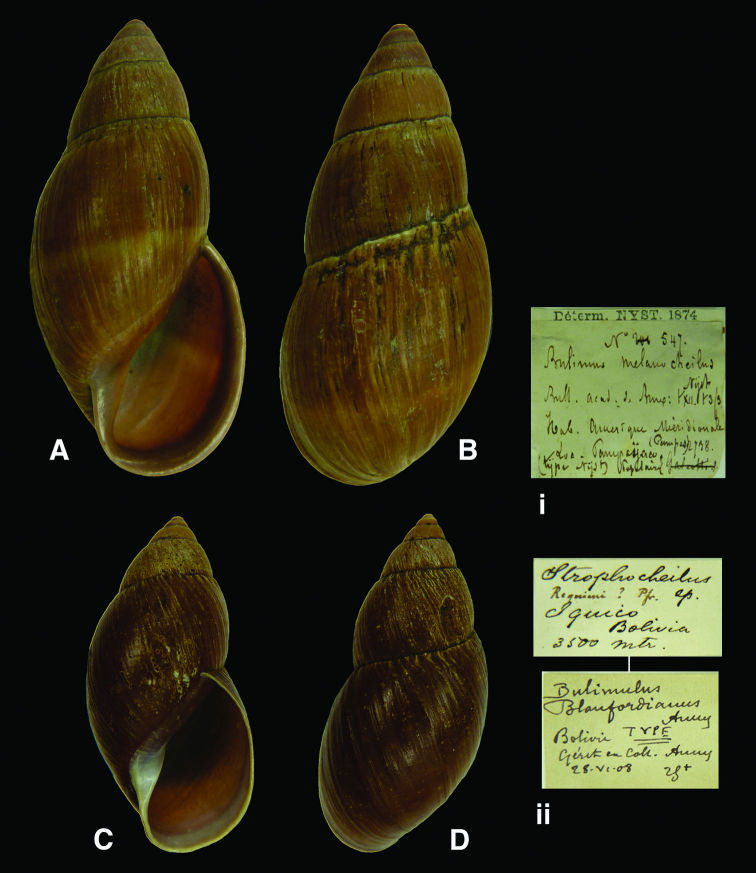
**A–B, i** *Thaumastus (Thaumastus) melanocheilus* (Nyst, 1845), lectotype RBINS/MT2361 (H=78.5). **C–D, ii** *Thaumastus (Thaumastus) blanfordianus* (Ancey, 1903), lectotype RBINS/MT1865 (H=52.5).

**Figure 5.**
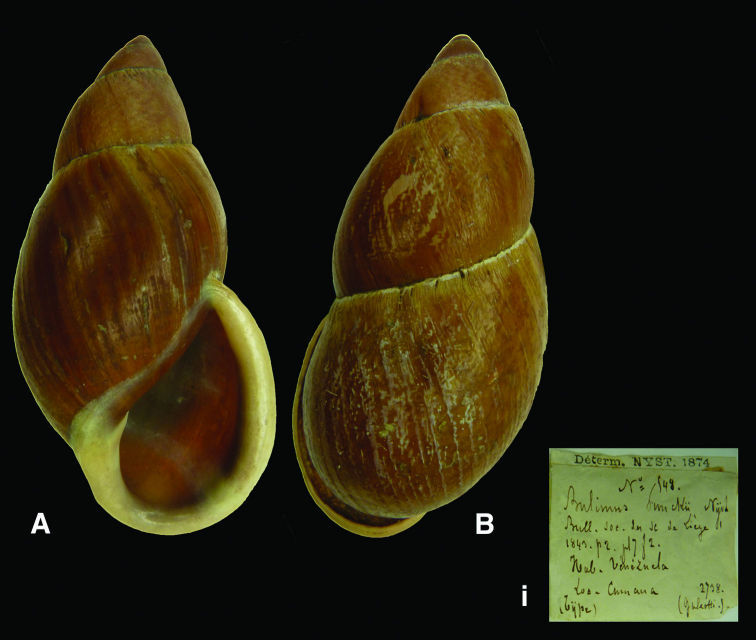
**A–B, i** *Dryptus funckii* (Nyst, 1843), lectotype RBINS/MT2352 (H=86.3).

**Figure 6.**
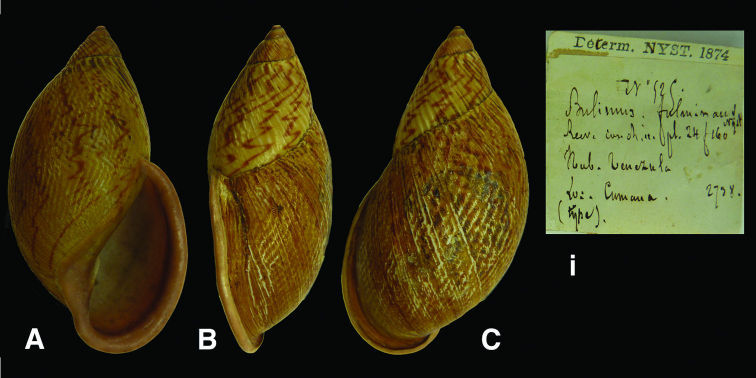
**A–C, i** *Plekocheilus (Plekocheilus) fulminans* (Nyst, 1845), lectotype RBINS/MT2351 (H=59.2).

**Figure 7.**
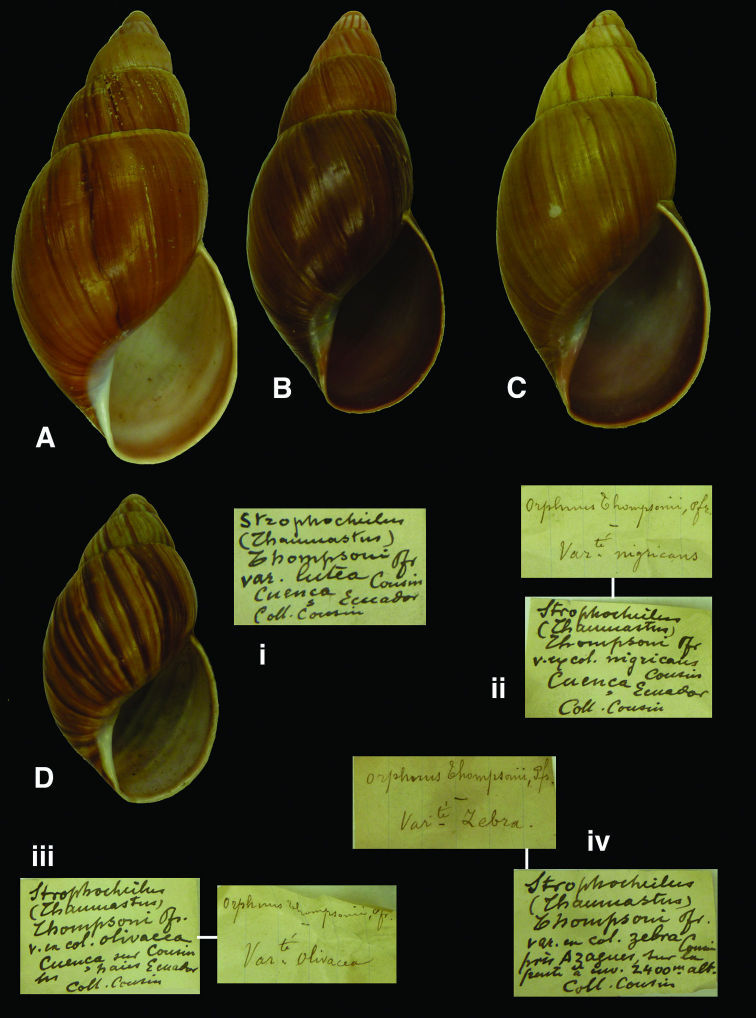
**A–D** *Kara thompsonii* (Pfeiffer, 1851); **A, i** lectotype var. *lutea* Cousin, 1887 RBINS/MT2358  (H=77.6); **B, ii** lectotype of var. *nigricans* Cousin, 1887 RBINS/MT2363 (H=62.8); **C, iii** lectotype of var. *olivacea* Cousin, 1887 RBINS/MT2366 (H=64.5); **D, iv** lectotype of var. *zebra* Cousin, 1887 RBINS/MT2375 (H=46.4).

**Figure 8.**
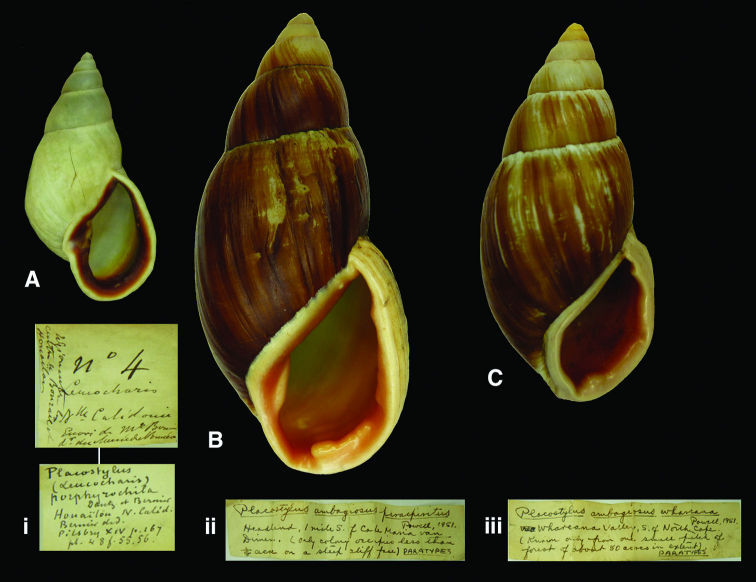
**A, i** *Aspastus porphyrochilus* (Dautzenberg and Bernier, 1901), syntype RBINS/MT2371  (H=41.9). **B–C** *Placostylus ambagiosus* (Suter, 1906) **B, ii** paratype of *Placostylus ambagiosus paraspiritus* Powell, 1951 RBINS/MT1969 (H=82.2). **C, iii** paratype of *Placostylus ambagiosus whareana* Powell, 1951 RBINS/MT376 (H=65.9).

**Figure 9.**
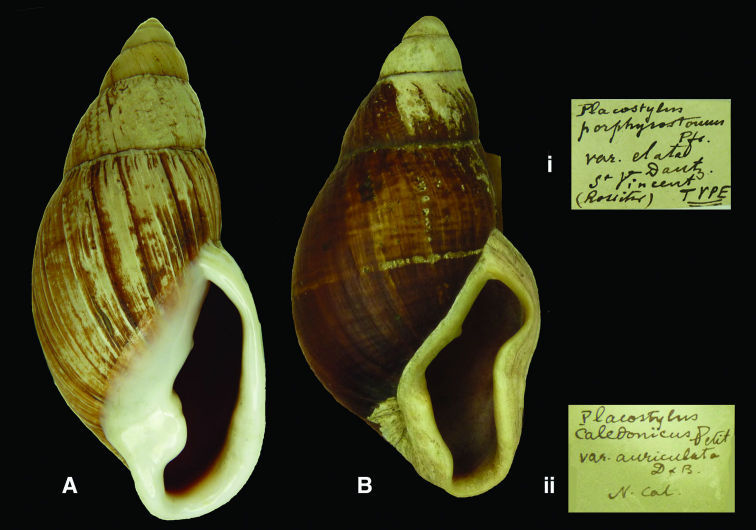
**A–B** *Placostylus*  **A, i** lectotype of *Placostylus elata* Dautzenberg, 1923 RBINS/MT702  (H=86.3) **B, ii** *Placostylus auriculata* Dautzenberg, 1923 RBINS/MT2339 (H=81.7).

**Figure 10.** *Bostryx* species. **A–B, i** *Bostryx alausiensis* (Cousin, 1887), paralectotype RBINS/MT2333  (H=25.2). **C–D, ii** *Bostryx juana* (Cousin, 1887), paralectotype RBINS/MT2357 (H= 19.1). **E, iii** *Bostryx chacoensis* (Preston, 1907), syntype RBINS/MT2343 (H=30.1) **F–G, iv** *Bostryx borellii* (Ancey, 1897), syntype RBINS/MT/xx (H=28.8). **H, v** *Bostryx carandaitiensis* (Preston, 1907), syntype RBINS/MT2341 (H=32.1).

**Figure 11.**
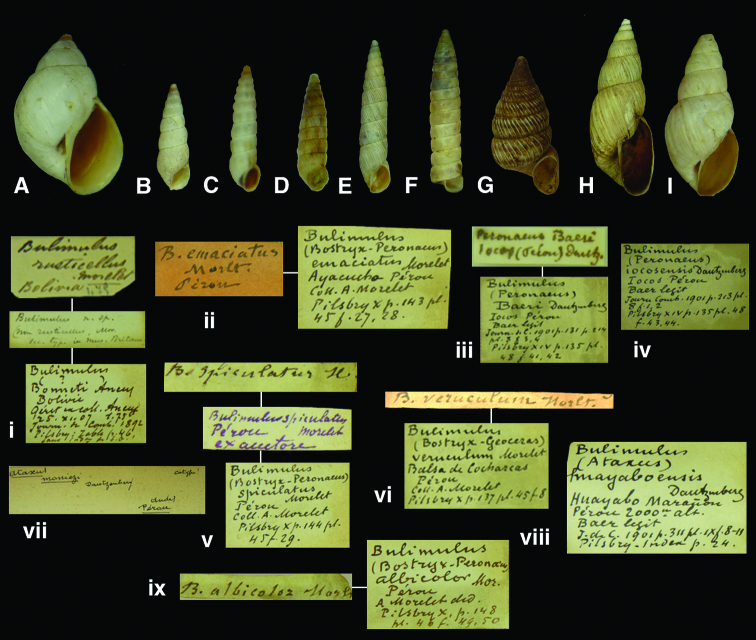
*Bostryx* species. **A, i** *Bostryx bonneti* (Ancey, 1902), paralectotype RBINS/MT2338 (H=21.9). **B, ii** *Bostryx emaciatus* (Morelet, 1863), paralectotype RBINS/MT2349 (H=18.3). **C, iii** *Bostryx baeri* (Dautzenberg, 1901), paralectotype RBINS/MT2336 (H=13.6). **D, iv** *Bostryx iocosensis* (Dautzenberg, 1901), paralectotype RBINS/MT2355 (H=11.3). **E, v** *Bostryx spiculatus* (Morelet, 1860), probable syntype RBINS/MT2373  (H=21.3). **F, vi** *Bostryx veruculum* (Morelet, 1860), probable syntype RBINS/MT2374 (H=21.9). **G, vii** *Bostryx moniezi* (Dautzenberg, 1901), syntype RBINS/MT1830 (H=13.9). **H, viii** *Bostryx huayaboensis* (Dautzenberg, 1901), paralectotype RBINS/MT2354 (H=22.9). **I, ix** *Bostryx orophilus* (Morelet, 1860), syntype of *albicolor* Morelet, 1863 RBINS/MT2335 (H=22.2).

**Figure 12.** *Drymaeus* species. **A, i** *Drymaeus (Drymaeus) abruptus* (Rolle, 1904), paralectotype RBINS/MT2332 (H=36.6). **B, ii** *Drymaeus (Drymaeus) nystianus* (Pfeiffer, 1853), lectotype of *nigricans* Cousin, 1887 RBINS/MT2365 (H=37.3). **C, iii** *Drymaeus (Drymaeus) colimensis* (Rolle, 1904), paralectotype RBINS/MT2344 (H=29.1). **D, iv** *Drymaeus (Drymaeus) poecilus ictericus* (Ancey, 1892), syntype RBINS/MT1881 (H=28.1). **E–F** *Drymaeus (Mesembrinus) granadensis* (Pfeiffer, 1848) **E, v** syntype of *interruptus* Preston, 1909 RBINS/MT2257 (H=22.6). **F, vi** syntype of *pallidus* Preston, 1909 RBINS/MT2258 (H=24.0).

**Figure 13.**
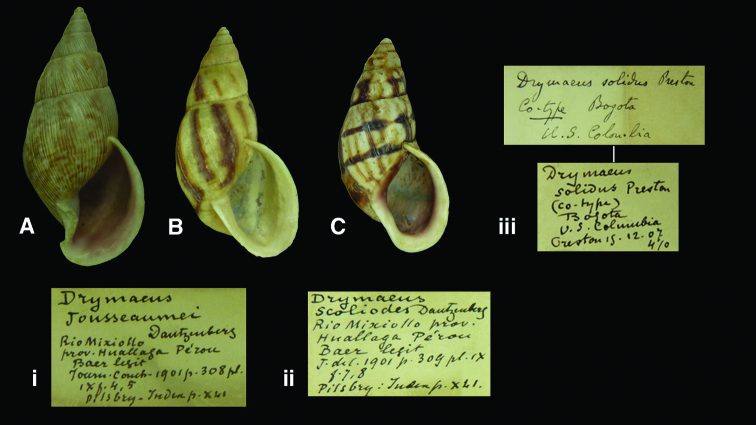
*Drymaeus* species. **A, i** *Drymaeus (Drymaeus) jousseaumei* (Dautzenberg, 1901), paralectotype RBINS/MT2356 (H=47.6). **B, ii** *Drymaeus (Drymaeus) scolioides* (Dautzenberg, 1901), paralectotype RBINS/MT2377 (H=40.7). **C, iii** *Drymaeus (Drymaeus) solidus* (Preston, 1907), syntype RBINS/MT2259 (H=33.8).

**Figure 14.**
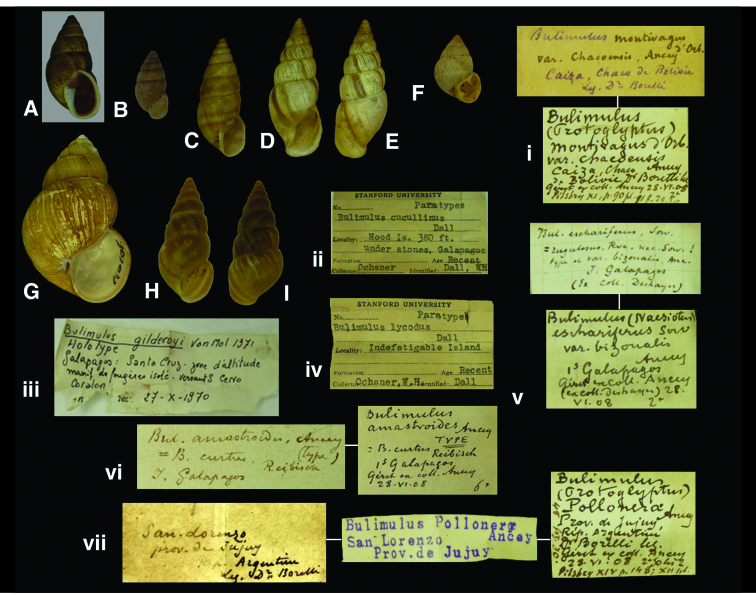
*Naesiotus* species. **A, ii** *Naesiotus cucullinus* (Dall, 1917), paratype RBINS/MT1833  (H=17.7). **B, vi** *Naesiotus amastroides* (Ancey, 1887), lectotype RBINS/MT1866 (H=9.25). **C, v** *Naesiotus eschariferus* (Sowerby, 1833), syntype of *Bulimulus eschariferus bizonalis* Ancey, 1887 RBINS/MT2337 (H=16.0). **D–E, i** *Naesiotus montivagus* (d’Orbigny, 1835), lectotype of *Bulimulus montivagus chacoensis* Ancey, 1897 RBINS/MT2342 (H=20.9). **F, iv** *Naesiotus lycodus* (Dall, 1917), paratype RBINS/MT1834 (H=11.4). **G, iii** *Naesiotus cavagnaroi* A.G. Smith, 1972, holotype of *Bulimulus gilderoyi* Van Mol, 1972 RBINS/MT106 (H=25.4). **H–I, vii** *Naesiotus pollonerae* (Ancey, 1897), lectotype RBINS/MT2369 (H=15.3).

**Figure 15.**
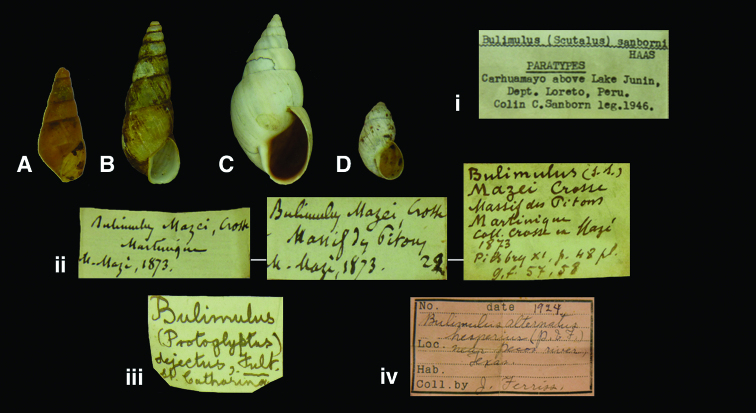
**A, ii** *Protoglyptus mazei* (Crosse, 1874), paralectotype RBINS/MT2360 (H=14.5). **B, iii** *Protoglyptus dejectus* (Fulton, 1907), paralectotype RBINS/MT2348 (H=28.0). **C, iv** *Rabdotus dealbatus* (Say, 1821), probable paratype of *Bulimulus alternatus hesperius* Pilsbry and Ferriss, 1924 RBINS/MT2353 (H=34.2). **D, i** *Kuschelenia (Vermiculatus) sanborni* (Haas, 1947), paratype RBINS/MT2372  (H=10.0).

**Figure 16.**
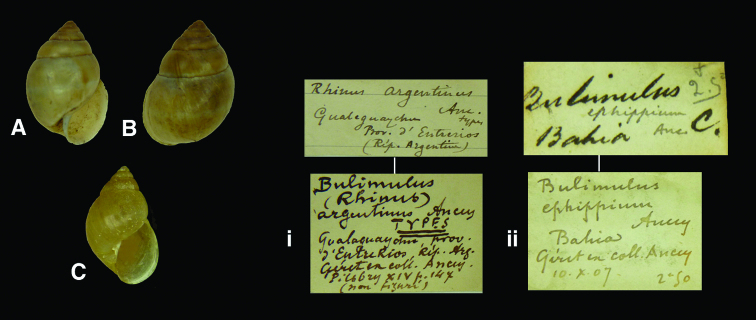
**A–B, i** *Rhinus argentinus* (Ancey, 1901), syntype RBINS/MT1867 (H=19.7).  **C, ii** *Simpulopsis (Eudioptus) ephippium* (Ancey, 1904), lectotype RBINS/MT2350 (H=19.5).

## New species

**Familiy Bulimulidae Tryon, 1867**

### Stenostylus

Pilsbry, 1898

http://species-id.net/wiki/Stenostylus

#### Remarks.

*Stenostylus* is a genus of high-altitude species, known to occur above 3000 m (Breure 1978), in Peru, Ecuador, and Colombia. It may be regarded as a sister-group of *Drymaeus* Albers, 1850 (Breure 1979: fig. 177).

#### Key to known species

**Table:** 

1	Surface with strong, thickened growth striae and traces of spiral impressions, shell height above 50 mm	2
–	Surface of shell relatively smooth, shell height up to 50 mm	3
2(1)	Last whorl relatively stout, aperture ovate; Peru, western slopes of Andes	*Stenostylus zilchi* Weyrauch, 1956
–	Last whorl relatively elongated, aperture elongate-ovate; Peru, eastern slopes of Andes	*Stenostylus perturbatus* sp. n.
3(1)	Spire pointed, whorls relatively flat; Peru, western slopes of Andes	*Stenostylus meleagris* (Pfeiffer, 1854)
–	Spire obtuse, whorls rounded	4
4(3)	Shell thin, smooth, shell height up to 20 mm; (?Colombia), Ecuador, eastern slopes of Andes	*Stenostylus colmeiroi* (Hidalgo, 1872)
–	Shell rather solid, with thickened growth striae, shell height more than 20 mm; Colombia, Cordillera Oriental	*Stenostylus nigrolimbatus* (Pfeiffer, 1854)

### Stenostylus perturbatus sp. n.

urn:lsid:zoobank.org:act:D4D62ABB-D38C-458C-99CD-506CC09E3BB3

http://species-id.net/wiki/Stenostylus_perturbatus

Figs 17A–B 

#### Diagnosis.

A large species of *Stenostylus*, with a very elongated spire, a roughly sculptured surface, and the aperture relatively small, and narrow, compared to other species of the genus.

#### Description.

Shell 56.5 mm, 2.0 times as long as wide, elongate, with straight sides, imperforate; rather thin. Colour dark-brown to blackish, with axially oriented, yellowish lines, varying from small to somewhat broader, in some places forming patches, but always irregular, never from suture to suture; upper whorls light-brown, the first ones denuded of the periostracum. Surface rather shining, with thickened growth striae where the colour is yellowish; some traces of spiral impressions, especially visible at the upper side of the last whorl. Protoconch eroded. Whorls 6.2, slight convex, the last 0.75 total shell height; suture well impressed. Aperture elongate-ovate, 0.45 times shell height, 1.49 times as long as wide, somewhat shining inside, whitish. Peristome thin, hardly expanded; columellar margin thin, curved, transitioning into the parietal wall, which has a very thin, whitish callus.

**Figure 17.**
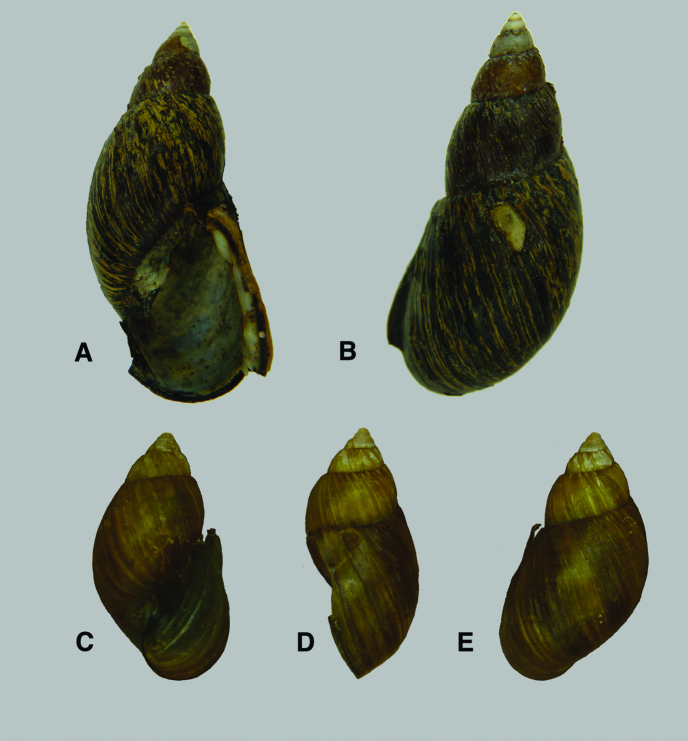
**A–B** *Stenostylus perturbatus* spec. nov., holotype RBINS/MT698 (H=56.5). **C–E** *Suniellus adriani* spec.nov., holotype RBINS/MT2378 (H=32.5).

#### Dimensions.

Holotype H 56.5, D 27.7, HA 25.5, WA 17.1.

#### Type locality.

Peru, Dept. Pasco, Huancabamba.

#### Type material.

RBINS/MT698, holotype (ex Preston).

#### Comparisons with other taxa.

This new species is placed in *Stenostylus*, despite its protoconch sculpture being unknown, on account of its general shape, the thickened growth striae, and the traces of a pearly lustre inside the aperture (*cf*. Breure 2008: 248). It resembles *Stenostylus zilchi* Weyrauch, 1956, which occurs on the western side of the Cordillera in Dept. Lima, and which differs from *Stenostylus perturbatus* by (1) being smaller; (2) having the last whorl more inflated; (3) having a wider aperture.

#### Remarks.

The single specimen known was found under the same manuscript name of Preston, from whom Dautzenberg obtained the shell on 30.xii.1909. The lip is damaged, especially at the columellar and basal side, and has partly been broken off.

#### Etymology.

(L.), *perturbatus*, unquiet; referring to the distinctive colour pattern of the shell. The epithet is used as an adjective.

### Suniellus

Breure, 1978

http://species-id.net/wiki/Suniellus

#### Remarks.

Hitherto this taxon was regarded a subgenus of *Scutalus* Albers, 1860. The latter genus appears to be polyphyletic (Breure, unpublished data); *Suniellus* is now interpreted as the high-altitude sister-group of *Kuschelenia* Hylton Scott, 1951 (cf. Breure 1979: fig. 172a), analogous to the relationship between *Stenostylus* and *Drymaeus*. There are currently four *Suniellus* species known, which are relatively undifferentiated in their external morphology; their vertical distribution extends 2600–4100 m (Breure 1979). These species are known from Peru and Bolivia (Breure 1978, 1979); the new taxon described herein thus extends the distribution range further north. In shell morphology the species may be confused with those of *Stenostylus*, but the protoconch sculpture separates the two taxa: a grating sculpture in *Stenostylus*; axial wrinkles, which may be partly anastomosing, in *Suniellus*.

#### Key to species

**Table:** 

1	Last whorl saccate, aperture elongate-ovate	2
–	Last whorl regularly rounded, aperture ovate	3
2(1)	Relatively small (up to 16 mm); Bolivia	*Suniellus chillu* Breure, 1978
–	Relatively large (more than 25 mm); Ecuador	*Suniellus adriani* sp. n.
3(1)	Shell height more than 25 mm; Colombia	*Suniellus goudoti* (Petit, 1843)
–	Shell height less than 25 mm; Peru	*Suniellus troscheli* (Philippi, 1867)

### Suniellus adriani sp. n.

urn:lsid:zoobank.org:act:B2AF1109-5F1F-41CE-A02C-4A7308AFF2D7

http://species-id.net/wiki/Suniellus_adriani

Figs 17C–E 

#### Diagnosis.

A relatively large, and elongate species of *Suniellus*, with the last whorl a little saccate.

#### Description.

Shell 32.5 mm, 1.86 times as long as wide, elongate-ovate, with hardly convex sides, imperforate; very thin. Colour yellowish-olivaceus with brownish streaks, especially on last whorl. Upper whorls lighter in colour. Surface rather shining, with irregularly spaced, thickened growth striae, in between with weaker incrassations and, especially at upper part of last whorl, some oblong granules. Protoconch eroded, but on the dorsal side traces visible of axial wrinkles, partly anastomosing. Whorls 5.7, hardly convex, the last 0.81 times total shell height, a little saccate; suture well impressed. Aperture elongate-ovate, 0.54 times shell height, approx. 1.4 times as long as wide, with a pearly lustre inside. Peristome thin and simple, columella thread-like, somewhat dilated above and appressed at the transition to the parietal wall, which bears a hardly noticeable callus.

#### Dimensions.

Holotype H 32.5, D 17.4, HA 17.4, WA 12.2.

#### Type locality.

Ecuador, Prov. Pichincha, “San Diego Cuchu” (see remarks).

#### Type material.

RBINS/MT2378, holotype, ex Cousin (Dautzenberg coll.).

#### Comparison with other taxa.

This novelty closely resembles *Suniellus chillu* Breure, 1978 from Bolivia in shell shape, but is much larger.

#### Remarks.

The lip is damaged at the upper palatal side and the insertion to the shell has been torn off, leaving some tears at the upper part of the last whorl, just behind the lip. The type locality could not be found in modern gazetteers; it is a mixture of Spanish and Quechua words, and may not be officially known under this name. It is likely to be in the páramo area, as Cousin on a second label has written “pie de la nieve / Frutillas por arriba” [at the foot of the snow / Strawberries above]; snow in the 19th century probably occurred at lower elevations than today, but an elevation for this locality of above 2750 m may be a safe guess (González, pers. commun.). There is only one specimen known.

#### Etymology.

The epithet is a patronym in honour of Adrián González—a Cuban malacologist now living in Quito, Ecuador—in recognition for his contributions to Neotropical malacology by his photographic work, his books aimed at a general public, and his dedicated fieldwork.

## Supplementary Material

XML Treatment for Bulimulus (Drymaeus) abruptus

XML Treatment for Thaumastus alausiensis

XML Treatment for Bulimulus (Naesiotus) albemarlensis

XML Treatment for Bulimus albicolor

XML Treatment for Bulimulus (Naesiotus) amastroides

XML Treatment for Bulimulus (Rhinus) argentinus

XML Treatment for Placostylus (caledonicus) auriculata

XML Treatment for Peronaeus baeri

XML Treatment for Bulimulus eschariferus bizonalis

XML Treatment for Bulimulus blanfordianus

XML Treatment for Bulimulus bonneti

XML Treatment for Bulimulus borellii

XML Treatment for Bulimulus (Drymaeus) carandaitiensis

XML Treatment for Bulimulus montivagus chacoensis

XML Treatment for Bulimulus (Drymaeus) chacoensis

XML Treatment for Otostomus colimensis

XML Treatment for Bulimus coloratus

XML Treatment for Bulimulus (Naesiotus) cucullinus

XML Treatment for Plecochilus dalmasi

XML Treatment for Bulimulus (Protoglyptus) dejectus

XML Treatment for Placostylus porphyrostomus elata

XML Treatment for Bulimus emaciatus

XML Treatment for Bulimulus ephippium

XML Treatment for Bulimus fulminans

XML Treatment for Bulimus funckii

XML Treatment for Bulimulus gilderoyi

XML Treatment for Bulimulus alternatus hesperius

XML Treatment for Bulimulus (Ataxus) huayaboensis

XML Treatment for Bulimus poecilus icterica

XML Treatment for Bulimulus (Drymaeus) interruptus

XML Treatment for Peronaeus (Peronaeus) iocosensis

XML Treatment for Drymaeus jousseaumei

XML Treatment for Thaumastus juana

XML Treatment for Orphnus thompsoni lutea

XML Treatment for Bulimulus (Naesiotus) lycodus

XML Treatment for Bulimulus mazei

XML Treatment for Bulimus melanocheilus

XML Treatment for Bostryx metagyra

XML Treatment for Bulimulus (Bostryx) moniezi

XML Treatment for Orphnus thompsoni nigricans

XML Treatment for Thaumastus nystianus nigricans

XML Treatment for Orphnus thompsoni olivacea

XML Treatment for Bulimulus (Drymaeus) interruptus pallidus

XML Treatment for Placostylus (Maoristylus) ambagiosus paraspiritus

XML Treatment for Bulimulus (Ataxus) perforatus

XML Treatment for Bulimulus pollonerae

XML Treatment for Leucocharis porphyrochila

XML Treatment for Bulimulus (Scutalus) sanborni

XML Treatment for Drymaeus scoliodes

XML Treatment for Bulimulus (Drymaeus) solidus

XML Treatment for Bulimus spiculatus

XML Treatment for Bulimulus (Protoglyptus) subcostatus

XML Treatment for Bulimus veruculum

XML Treatment for Placostylus (Maoristylus) whareana

XML Treatment for Orphnus thompsoni zebra

XML Treatment for Stenostylus

XML Treatment for Stenostylus perturbatus

XML Treatment for Suniellus

XML Treatment for Suniellus adriani
